# Alkyl-Substituted Aminobis(phosphonates)—Efficient Precipitating Agents for Rare Earth Elements, Thorium, and Uranium in Aqueous Solutions

**DOI:** 10.1021/acsomega.1c02982

**Published:** 2021-09-13

**Authors:** Emilia J. Virtanen, Siiri Perämäki, Kaisa Helttunen, Ari Väisänen, Jani O. Moilanen

**Affiliations:** † Department of Chemistry, Nanoscience Center, University of Jyväskylä, P.O. Box 35, FI-40014 Jyväskylä, Finland; ‡ Department of Chemistry, University of Jyväskylä, P.O. Box 35, FI-40014 Jyväskylä, Finland

## Abstract

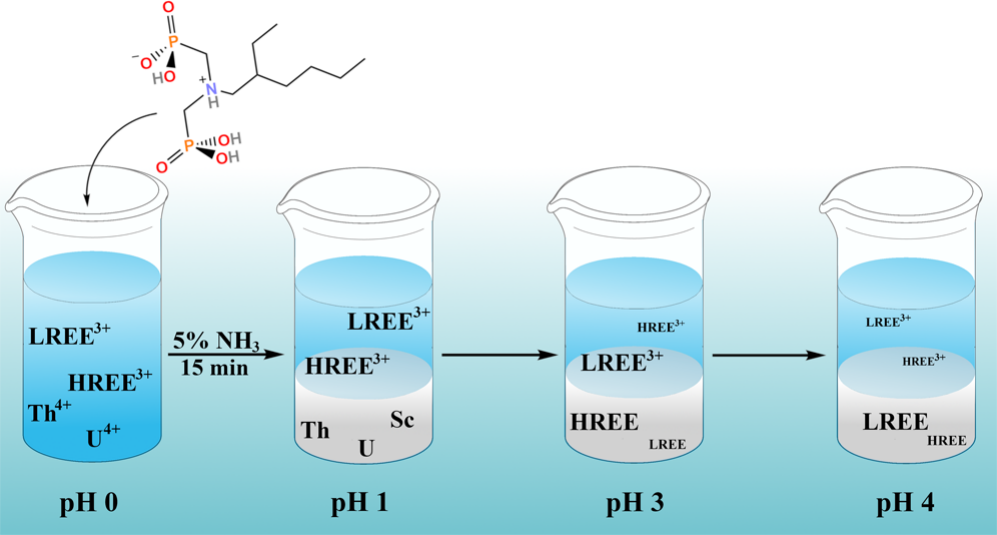

The efficient and environmentally sustainable separation process for rare earth elements (REE), especially for adjacent lanthanoids, remains a challenge due to the chemical similarity of REEs. Tetravalent actinoids, thorium, and traces of uranium are also present in concentrates of REEs, making their separation relevant. This study reports six simple water-soluble aminobis(phosphonate) ligands, RN[CH_2_P(O)(OH)_2_]_2_ (**1** R = CH_2_CH_3_, **2** R = (CH_2_)_2_CH_3_, **3** R = (CH_2_)_3_CH_3_, **4** R = (CH_2_)_4_CH_3_, **5** R = (CH_2_)_5_CH_3_, **6** R = CH_2_CH(C_2_H_5_)(CH_2_)_3_CH_3_) as precipitating agents for REEs, Th, and U, as well as gives insight into the coordination modes of the utilized ligands with REEs at the molecular level. Aminobis(phosphonates) **4**–**6** with longer carbon chains were found to separate selectively thorium, uranium, and scandium from REEs with short precipitation time (15 min) and excellent separation factors that generally range from 100 to 2000 in acidic aqueous solution. Ligands **1**–**6** also improved separation factors for adjacent lanthanoids in comparison to traditional oxalate precipitation agents. Importantly, precipitated metals can be recovered from the ligands with 3 molar HNO_3_ with no observed ligand decomposition enabling the possibility of recycling the ligands in the separation process. NMR-monitored pH titrations for **1** showed deprotonation steps at p*K*_a_ 1.3, 5.55, and >10.5, which indicate that the ligands remain in a deprotonated [**L**]^−1^ form in the pH range of 0–4 used in the precipitation studies. ^31^P NMR titration studies between **1** and M(NO_3_)_3_ (M = Y, La, Lu) gave satisfactory fits for 1:3, 1:2, and 1:1 metal–ligand stoichiometries for Y, La, and Lu, respectively, according to an F-test. Therefore, aminobis(phosphonate) precipitation agents **1**–**6** are likely to form metal complexes with fewer ligands than traditional separation agents like DEHPA, which coordinates to REEs in 1:6 metal–ligand ratio.

## Introduction

Rare earth elements (REE) consisting of lanthanoids, scandium, and yttrium are widely used in crucial technological applications such as computers, catalysts, batteries of electric cars, and renewable energy production; latter two play an important role in a shift toward greener technologies.^1^ Thus, the demand of REEs has been estimated to increase considerably in the future. In EU alone, e-mobility and renewable energy production could increase the demand for dysprosium and neodymium up to 12- and 4-fold by 2050 from the current demand of 200 and 4000 tons, respectively.^2^ Globally, the demand for all REEs has been estimated to grow annually 4.4% until 2026, raising concerns for the sufficiency of primary production of REEs from ores, which is not an environmentally sustainable process (see below).^3^ Ores of REEs also contain radioactive elements like thorium and uranium that complicate the separation process of REEs.^4^ Therefore, it is not only important to investigate the recycling and recovery of REEs from secondary sources, where the concentration of REEs is relatively high,^5−7^ but also to develop new separation processes for REE concentrates that allow the efficient and environmentally friendly separation of REEs from each other and other metals.

The most common separation process for lanthanoids includes liquid–liquid extraction with organophosphorous extracting agents, while Th and U are typically separated from lanthanoids first by selective dissolution and further purificated by liquid–liquid extraction.^4^ The most commonly used extracting agents for lanthanoids are di(2-ethylhexyl)phosphoric acid (DEHPA) and 2-ethylhexylphosphonic acid mono-2-ethylhexyl ester (EHEHPA) due to their robustness and good recyclability, whereas Th and U can be separated from REEs, and further from each other using tributylphosphate (TBP) or secondary and tertiary amines.^8^ However, lanthanoids are chemically a very similar group of elements, and especially the separation of adjacent lanthanoids is challenging even with the commercial extracting agents. Although a liquid–liquid extraction process is the most suitable for industrial scale, one of the challenges has been to reduce the amount of used organic solvents to make the process more sustainable.^4^ The liquid–liquid extraction utilizing commercial extraction agents, such as DEHPA and EHEHPA, can be improved by replacing organic solvents with ionic liquids.^9−13^ This replacement has shown improvements in the separation of heavy and light adjacent lanthanoids,^9,10^ selectivity to Nd over transition metals,^11^ and high La/Ce separation.^12^ Ionic liquids have also been used in Th/U separation with a success as very good separation factor is obtained (SF_Th/U_ 793).^13^ However, the high viscosity of ionic liquids still remains a challenge in the extraction processes.

Apart from the liquid–liquid extraction, separation for REEs and Th can also be done solely in water solution with no need for the organic phase, either by precipitation or fractional crystallization. Traditional precipitation agents, oxalates, have been reported to separate light lanthanoids from heavy ones by selective dissolution of rare earth oxalates.^14^ Fractional crystallization from water solutions with borates^15^ or coordination polymers^16,17^ have yielded good separation factors, especially for Nd/Dy separation (SF_Nd/Dy_ > 300). Furthermore, selective crystallization with the iodate–sulfate system has been reported to separate efficiently lighter lanthanoids from heavier ones,^18^ and selenite crystallization has yielded good Th/Ln separations.^19^ Nevertheless, the crystallization method with borates, iodate–sulfate, and selenite systems requires long reaction times of 5 days, hydrothermal conditions (>453 K), and in the case of borate systems, environmentally hazardous bromoform for the final separation step.^15^

Aminophosphonates have gathered attention in the medical field due to their pharmaceutical properties^20^ and good binding affinity toward medically relevant lanthanoids, such as gadolinium (common MRI contrasting agent) and samarium (nuclear medicine).^21^ Despite the good coordination properties of aminophosphonates toward REEs, their utilization in REE recovery and separation has been initiated only recently, yielding promising results. For example, the separation factors of (2-ethylhexylamino)methylphosphonic acid mono-2-ethylhexyl ester (HEHAMP) and 2-ethylhexyl-3-(2-ethylhexylamino)pentan-3-yl-phosphonic acid (HEHAPP) in the liquid–liquid extraction process of REEs are larger in comparison to the separation factors of two conventional extracting agents, DEHPA and EHEHPA.^22−26^ Similarly, tetravalent Th/Ce separation in a liquid–liquid extraction with an aminophosphonate-based extracting agent Cextrant 230 gives a good separation factor of 14.7.^27^

**Scheme 1:**
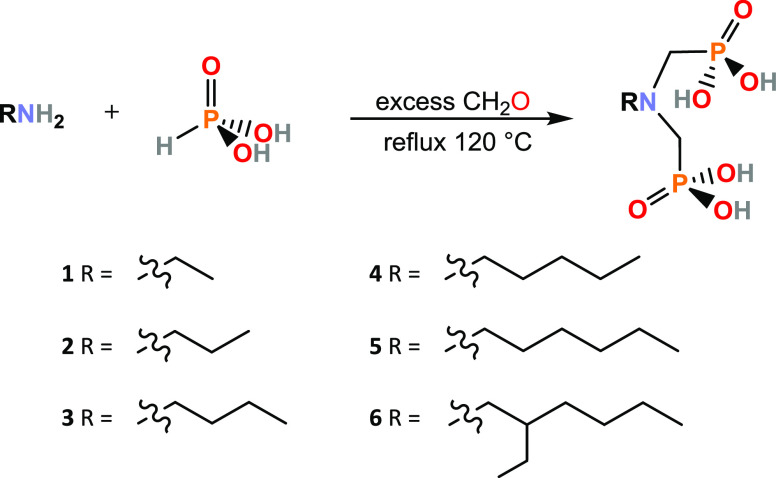
General Synthesis Route for Aminomethylphoshonate Ligands 1–6

Depending on the nature of organic moiety and the number of phosphonate groups in the aminophosphonate framework, aminophosphonates can be designed to be water soluble,^28^ which would enable their use as precipitation agents for metals in acidic aqueous solutions, similar to oxalates.^20^ Furthermore, by varying the number of phosphonate group and/or organic moiety, precipitation abilities of aminophosphonates toward different metal ions can be tuned. For example, by increasing the number of phosphonate groups in the ligand framework, more binding sites are available for metal ions in a single ligand. With that being said, we investigated the complexation and precipitation properties of simple aminobis(phosphonates) **1**–**6** (Scheme 1) toward REEs, Th and U in NMR, and larger (∼100 mg) scale in different pH values ranging from 1 to 4. We also determined the acid–base properties of synthesized aminobis(phosphonate) ligands utilizing NMR spectroscopy and carried out computational analysis for the most plausible complexes in the aqueous solution to get further insight into their solution behavior. To the best of our knowledge, this study demonstrates for the first time that simple aminobis(phosphonates), which can be synthesized by straightforward addition reactions, can be used as efficient precipitation agents with short precipitation times for REEs, Th, and U in aqueous solutions.

## Results and Discussion

Ligands **1**–**6** were prepared using a modified reported one-pot synthesis, where a condensation reaction between an amine and formaldehyde is followed by nucleophilic addition of phosphorous acid under reflux condition in an acidic water solution (Scheme 1).^28^ Crude products were purified by recrystallization either from ethanol, water, or water:ethanol (2:1) mixture. The purity of the recrystallized products was ensured by ^1^H NMR, IR, and elemental analysis (Figures S1–S12).

As expected, the ligands with shorter carbon chains (**1**–**3**) showed higher solubility in water compared to ligands with longer (**4** and **5**) and branched (**6**) chains (Table S1). For example, the water solubility of ligands **1**, **3**, and **6** were 327, 184, and 9.5 g/L, respectively.

### Acid–Base Properties

The deprotonation processes of the most water-soluble ligand **1** were investigated to assess the protonation state of ligands in metal complexes. Dependence of the deprotonation steps of **1** on the pH was determined by NMR titrations in D_2_O at 295 K. The pH of the 0.14 M solution of **1** was adjusted between 0.5 and 10.5 with the addition of a 5% NH_3_ solution, and ^31^P and ^1^H NMR spectra were measured at 0.5 pH unit intervals. Figure S13 shows the measured ^31^P NMR and ^1^H NMR shifts as a function of pH for the P atom and N–CH_2_–P protons, respectively. ^1^H NMR shifts of OH protons were not used in the determination since they cannot be directly observed in D_2_O due to the fast proton–deuterium exchange. Three equivalent points for the deprotonation steps of **1** were observed at pH 1, pH 5, and around pH 10. The observed shift for the first deprotonation is slightly different for ^31^P nucleus than for the ^1^H nuclei, but otherwise, the data obtained from ^31^P and ^1^H experiments are consistent. p*K*_a_ values for the first two deprotonations were calculated from the observed chemical shifts according to a previously reported method.^29^ In contrast, the third deprotonation step at 10.5 takes place at the end of the titration (pH of ammonia is ∼10.6). Thus, the p*K*_a_ could not be calculated for the third deprotonation step. For the first and second deprotonation steps, the calculated p*K*_a1_ and p*K*_a2_ values are 1.33 and 5.55, respectively.

**Scheme 2:**
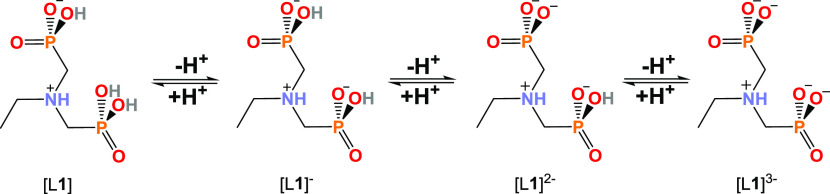
Structures of Ligand **1** (L1) after Each Deprotonation Step

The deprotonation steps of **1** can be further elaborated by comparing the determined p*K*_a_ values to the p*K*_a_ values of two polyprotic phosphoric acids, namely pyrophosphoric acid (p*K*_a1_ = 0.91, p*K*_a2_ = 2.10, p*K*_a3_ = 6.70, and p*K*_a4_ = 9.32)^30^ and pamidronic acid (p*K*_a1_ = 1.85, p*K*_a2_ = 5.85, and p*K*_a3_ = 10.30).^31^ Assuming that **1** exists as a zwitterion, the determined p*K*_a1_ value 1.33 suggests that the first deprotonation likely occurs from the fully protonated P(OH)_2_(O) group forming structure [L**1**]^−^ (Scheme 2), similar to pamidronic acid, which is known to exist as a zwitterion in a low pH regime.^32^ The zwitterionic nature of **1** is further supported by the fact that it has only one low p*K*_a_ value (<2.50), arising from the deprotonation of one of the P(OH)_2_(O) groups, in contrast to pyrophosphoric acid which has two low p*K*_a_ values due to the two fully protonated P(OH)_2_(O) groups. By comparing the p*K*_a2_ value (5.55) of **1** to the p*K*_a3_ (6.70) and p*K*_a2_ (5.85) values of pyrophosphoric acid and pamidronic acid, respectively, it can be concluded that the second deprotonation step originates from either of the P(OH)(O^–^) groups forming a twice deprotonated structure [L**1**]^2–^. The third deprotonation step takes place either from the P(OH)(O^–^) or the R_3_NH^+^ group. However, aminophosphonates have been reported to deprotonate first fully from the phosphorous groups before the NH^+^ deprotonation is observed to occur.^31,33^ Therefore, the third observed deprotonation is most likely to occur from the last P(OH)(O^–^) proton forming a structure [L**1**]_3_^–^ in pH > 10.5.

### Complexation Studies

The binding affinity of ligand **1** toward REEs was investigated by performing NMR titrations in D_2_O with three different metal salts—Y(NO_3_)_3_, La(NO_3_)_3_, and Lu(NO_3_)_3_—at low pH values (∼1.4–2.4), where **1** exists as a monoanion. These metal salts were chosen because of their different ionic radii and diamagnetic nature (no unpaired electrons). NMR titrations were also attempted for **1** with Sc(NO_3_)_3_ and Th(NO_3_)_4_ by adding 1 mM metal to 10 mM ligand. Unfortunately, Sc and Th complexes of **1** precipitated out from D_2_O during titrations even at low pH values, preventing further analysis of the titration data.

First, metal-to-ligand titrations were carried out for Y by adding incremental amounts of Y(NO_3_)_3_ into 10 mM solution of **1** in D_2_O. After each addition of the metal salt, ^31^P and ^1^H NMR spectra were measured. The ^31^P NMR spectrum of the free ligand displayed one triplet for the P atoms at 8.54 ppm, and the ^1^H spectrum showed doublet, quartet, and triplet for N–CH_2_–P, C–CH_2_–N, and CH_3_ protons, respectively. Since ^1^H resonances overlapped strongly in metal-to-ligand (Figure S14) and also in reverse ligand-to-metal titrations (see below and Figure S15), the chemical shift change of the ^31^P signal was followed. During the titration, the ^31^P signal shifted 2.54 ppm upfield, indicating that the free ligand and complexed species experienced fast exchange dynamics on the NMR timescale (Figure S16). Saturation of the chemical shift changes of phosphorus was observed after the addition of 1 equiv of metal. However, when **1** was titrated with La and Lu, smaller, <1 ppm upfield shift in ^31^P signal was observed without saturation of the chemical shift changes at 1 equiv of metal (Figures S17 and S18). Additionally, the precipitate was observed during Lu titration; therefore, pH was set to 1.0 to prevent Lu complex from precipitating.

**Figure 1:**
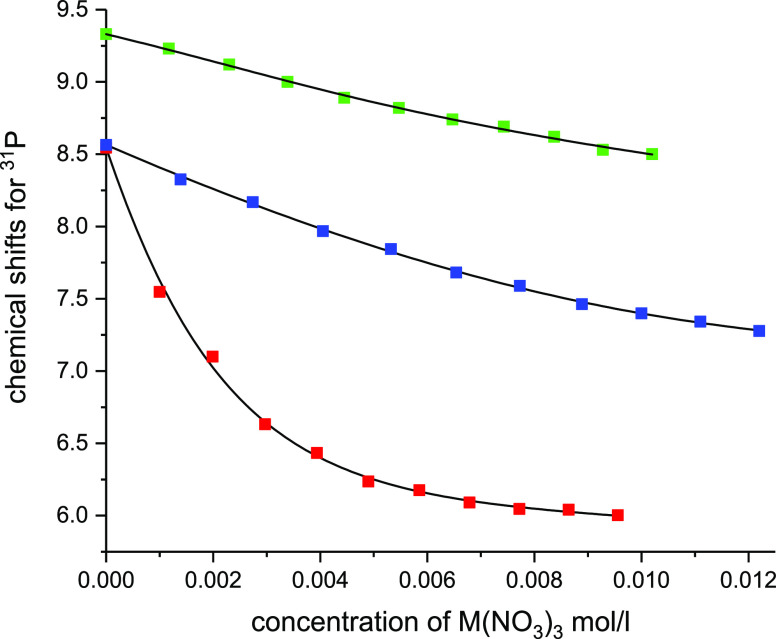
^31^P NMR shift changes as a function of the concentration of M(NO_3_)_3_ when ligand **1** is titrated with Y(NO_3_)_3_ (red) and La(NO_3_)_3_ at pH 1.8 (blue), and Lu(NO_3_)_3_ at pH 1.0 (green). Fittings for 1:3, 1:2, and 1:1 (M/L) binding models are presented for Y, La, and Lu, respectively (black solid lines).

For Y, analysis of the titration data to theoretical 1:1 and 1:2 (M/L) binding isotherms provided unsatisfactory fits with relatively large errors of fit (Figure S19), whereas the addition of a third binding constant K_3_ for the 1:3 binding model improved the fit significantly (Figure 1). Data was also fitted to a theoretical 1:4 binding isotherm (Figure S19). Because a small improvement of fit was observed by introducing more variables to the model, statistical F-tests were carried out for all fits to assign the preferential binding model. Based on the F-tests, the 1:3 binding model provided the best fit for the titration data at this range of concentrations for Y (Table S2). Similar fits to theoretical 1:1, 1:2, 1:3, and 1:4 binding isotherms were obtained for La as for Y (Figures 1 and S20); however, the F-tests indicated that the 1:2 binding model was slightly better than the 1:3 model (Table S3). Interestingly, for Lu, analysis of the titration data to a theoretical 1:1 binding isotherm already provided a satisfactory fit (Figures 1 and S20), which was confirmed by the F-test (Table S4). The binding constants obtained for the 1:1, 1:2, and 1:3 metal complexes (Table 1) indicate that all M/L complexes are being formed in the solution when the ligand is titrated with Y or La.

**Table 1:** Overall Logarithmic Binding Constants (log *K*) for 1:1, 1:2, and 1:3 (Metal:Ligand) Binding Models^a^

	log *K* 1:1	log* K* 1:2	log *K* 1:3
**Y**_**ML**_	2.4 ± 0.2	4.9 ± 0.4	7.3 ± 0.6
**Y**_**LM**_	2.6 ± 0.2	4.4 ± 0.4	6.7 ± 0.7
**La**_**ML**_	2.6 ± 0.5	4.4 ± 0.1	
**La**_**LM**_	2.7 ± 0.5	4.3 ± 0.4	6.8 ± 0.3
**Lu**_**ML**_	2.1± 0.3		
**Lu**_**LM**_	N.D.^b^		

a M_ML_ represents the metal-to-ligand titration and M_LM_ the ligand-to-metal titration (M = Y, La, Lu). Errors are derived from standard deviation.

b Sample formed a gel.

Reverse ligand-to-metal titrations were also performed by titrating 10 mM M(NO_3_)_3_ with incremental addition of **1**. However, the addition of 3 equiv of **1** to the NMR tube containing Lu(NO_3_)_3_ promoted the formation of a gel-like structure, thus preventing the determination of the binding constant for Lu. For Y and La, the first spectra were recorded after the addition of 0.3 eq of ligand, where phosphorus nuclei resonated at 6.25 and 7.02 ppm for Y and La, respectively (Figures S21 and S22). The titration was continued until the concentration of **1** reached 5 equiv, at which point, the chemical shift of the phosphorus signal had changed to 8.16 ppm for Y and 8.25 ppm for La without saturation of the chemical shift changes.

**Figure 2:**
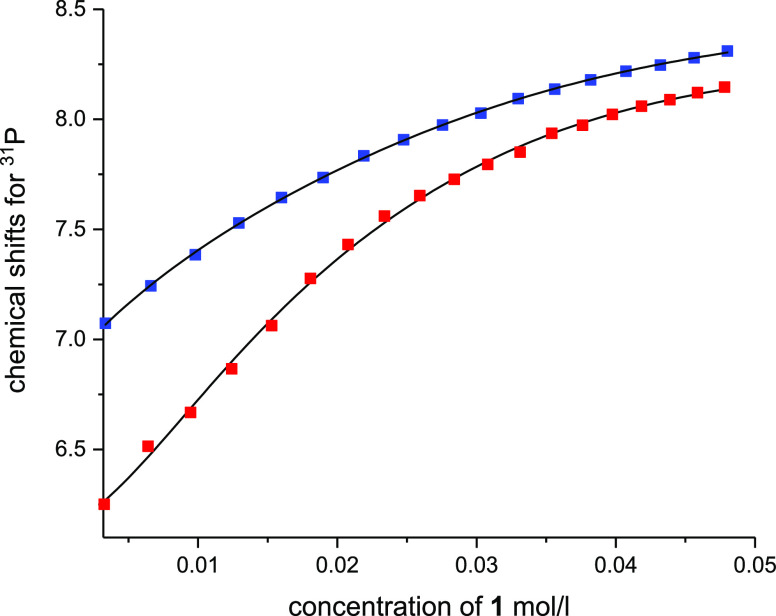
^31^P NMR shift change as a function of the concentration of **1** when Y(NO_3_)_3_ (red) and La(NO_3_)_3_ (blue) are titrated with **1**. Fittings for 1:3 (M/L) binding models are presented as black lines.

Analysis of the titration data to a 1:1 binding model provided unsatisfactory fits for both Y and La, whereas the addition of 1:2 and 1:3 binding models showed a slight improvement to the fits (Figures 2, S23, and S24). Similarly, to the metal-to-ligand titration, fourth binding constant K_4_ was also fitted to the titration data of Y and La where it provided the best fit for one of the repeats in both cases (Tables S5 and S6). Based on the unsaturation observed in the titration data and partial success in the fitting of the 1:4 model, it is possible that higher-order complexes are formed in the solution when the concentration of the ligand is high enough. Overall, the binding constants obtained from the reverse ligand-to-metal titrations are similar to the metal-to-ligand binding constants for Y and La, although small differences can be observed (Table 1). The differences most likely arise from the different forming order of the complexes in the titrations, as in the reverse ligand-to-metal titration, the 1:1 complex forms first followed by the 1:2 and 1:3 complexes.

**Scheme 3:**
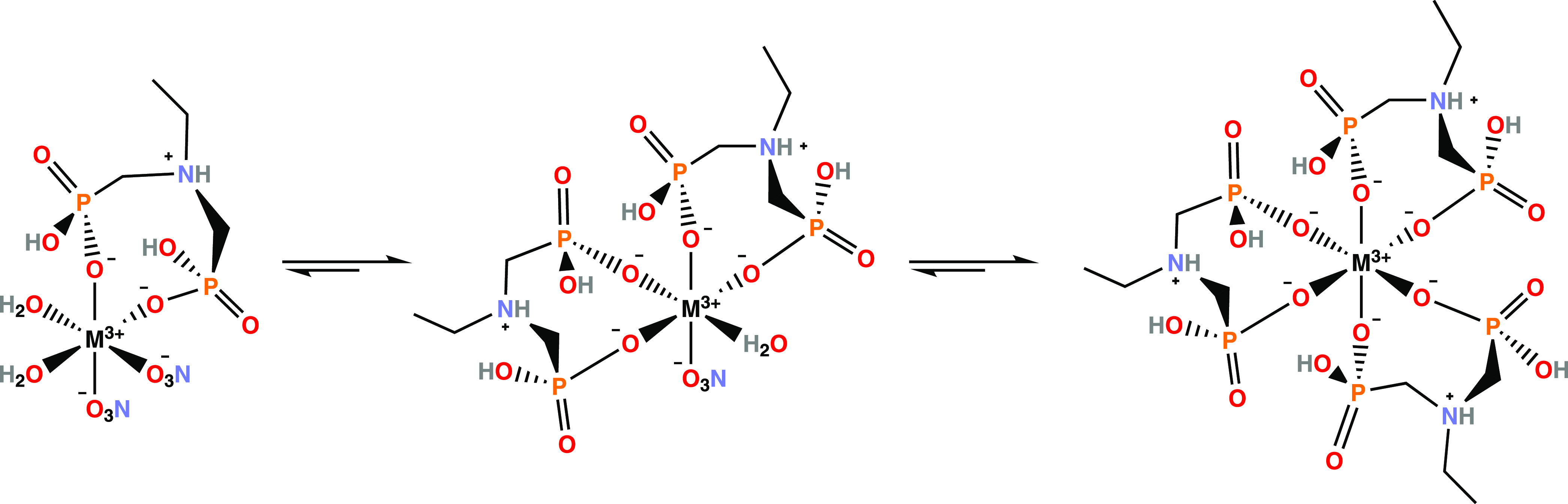
Proposed Zwitterionic Structures for 1:1, 1:2, and 1:3 M/L Metal Complexes (M = Y, La, Lu) with Ligand **1**

Proposed complexation structures based on the titrations for Y, La, and Lu complexes are illustrated in Scheme 3. As the pH was between 1.4 and 2.4 during titrations, **1** is expected to coordinate to the metals in the deprotonated [L**1**]^−1^ form (Scheme 2), which is the most likely form of **1** at the lower pH region. This protonation state also provides neutral 1:3 complexes.

Further insight into the coordination properties of ligands was obtained from the density functional theory calculations, which were carried out for the 1:3 complex of Y^3+^ and three [**L**]^−^ (**L** = CH_3_N[CH_2_P(O)(OH)_2_]_2_) in the neutral and zwitterionic form in the solution state. The calculations predicted the zwitterionic form to be significantly more stable (76 kJ mol^–1^) than the neutral one, which is consistent with the NMR studies. As illustrated with the space-filling model of the most stable optimized 1:3 complex, three [**L**]^−^ ligands do not entirely complete the coordination sphere of Y^3+^ (Figure S25). Thus, it is likely that in the aqueous solution coordinated water molecules and/or other species present in the solution might coordinate Y^3+^ ion in 1:3 complex. The results further support that 1:4 complexes cannot be fully ruled out due to steric reasons, although 1:3 complexes are the most likely species in the aqueous solution at least for the REEs with larger ionic radii based on the NMR studies.

The 1:3 M/L stoichiometry proposed for M:**1** complexes was compared with other phosphonate–metal complexes reported in the literature. The 1:3 metal–ligand stoichiometry has also been reported for the complexes of lanthanoids and nitrilotris(methylphosphonic acid), whereas commercial extraction agent DEHPA, which contains only one phosphonate group, forms 1:6 complexes with REEs and actinoids.^34−37^ Although NMR titration data with Th could not be analyzed, the literature suggests that bisphosphonates bind into Th and U either with similar 1:3 or 1:2 stoichiometry, and depending on the medium, nitrate or sulfate ions fulfill the coordination sphere.^38−42^ As uranium is commonly precipitated as an ammonium salt by injecting NH_3_ and CO_2_ gases into a uranium-containing solution, it is therefore highly possible for uranium to also form insoluble ammonium salts at higher pH values.^43,44^ Also, Th forms insoluble ammonium salts in the solution with higher pH (see below). Taken together, the obtained results indicate that a smaller amount of **1**–**6** is needed for the REE separation process compared to the commercial liquid–liquid extraction (DEHPA and EHEHPA), which form 1:6 complexes with REEs.^34−37^ On the other hand, commercial precipitation agents (oxalates) are needed in smaller quantities than ligands **1**–**6** since oxalates have been reported to bind with 2:3 metal–ligand ratio.^45,46^

### Precipitation Studies

The precipitation properties of ligands **1**–**6** toward REEs, Th, and U in 5% HNO_3_ solution were investigated in a pH range of 0–4. The pH was not increased above 4 to avoid precipitation of lanthanoid hydroxides.^47^ pH was set with 5% NH_3_ solution and the precipitation percentages were determined by taking into account the dilution of the added base, calculating the precipitated amount for each metal, and dividing the precipitated amount by the concentration at the beginning. As the expected stoichiometry for the metal–ligand ratio is 1:3, solutions were prepared with a 6-fold excess of the ligands to ensure sufficient amount of the precipitation agents. From each pH, the sample was taken aside, filtrated, and diluted with 5% HNO_3_ for the inductively coupled plasma optical emission spectrometer (ICP-OES) measurements. Precipitation studies were also performed without the presence of ligands to ensure that the metal complexes do not precipitate out from the solution as ammonia salts (Table S7). No precipitation or minimal precipitation was observed for REEs and U with ammonia, whereas Th precipitated out from the solution at pH higher than 2.5.

**Figure 3:**
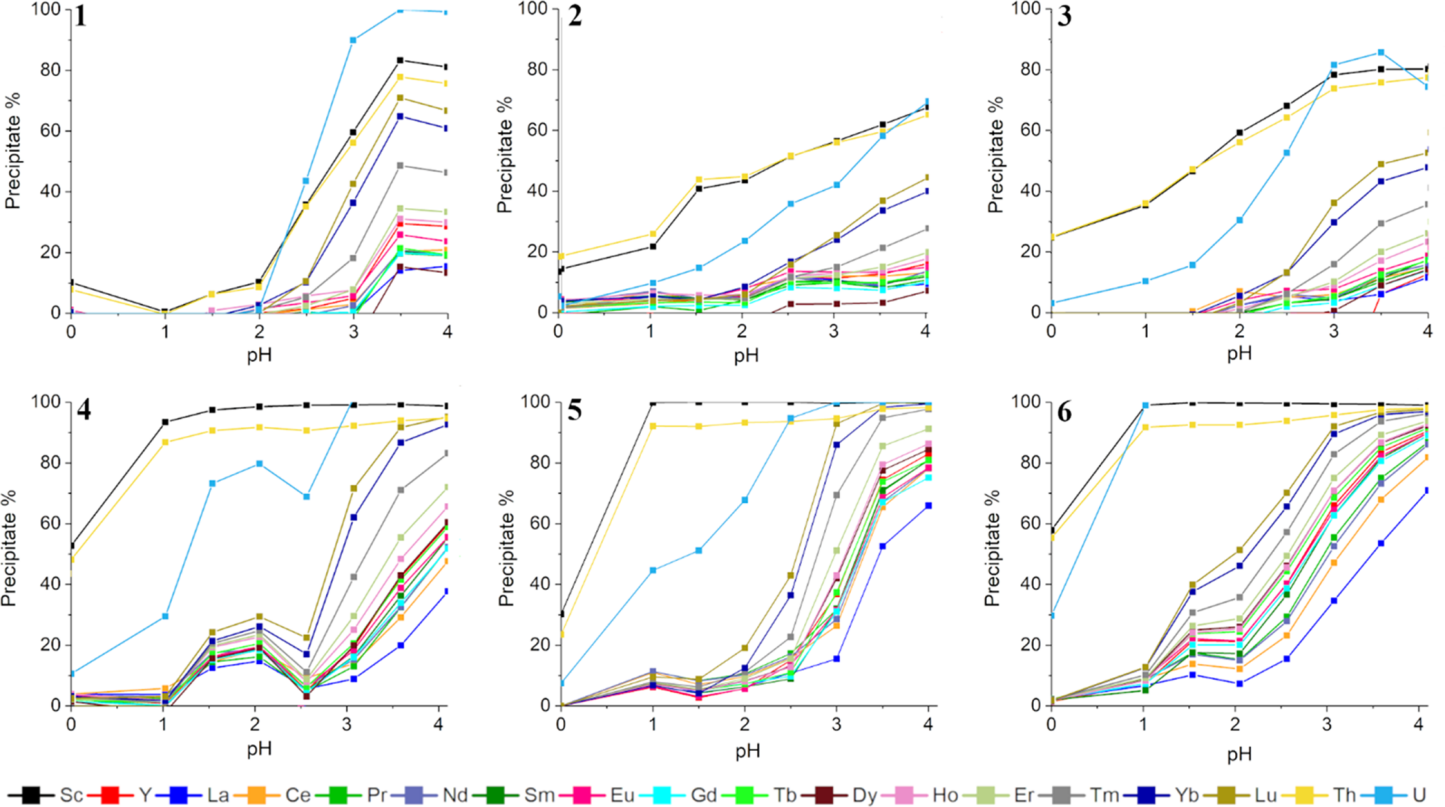
Precipitation percentages of REEs, Th, and U in different pH for ligands **1**–**6** (from the top left to the bottom right). For clarity, the error bars are omitted from the figure, but the standard deviation errors for precipitation percentages are given in Tables S8–S13.

Figure 3 shows the precipitated percentages for ligands **1**–**6** in the pH range of 0–4, and five main trends can be observed from it. First, the deprotonated form of ligand [**L**]^−^ increases when the pH of the solution increases, resulting in higher precipitation percentages of REEs, Th, and U. Second, ligands **4**–**6** with longer carbon chains precipitate more metals out from the solution than **1**–**3** with shorter carbon chains, with the exception of ligand **1**, which unexpectedly precipitates out more some of the metals (Er–Lu, Th, and U) than ligands **2** and **3**. Third, ligand **6** precipitates U, Th, and Sc selectively at pH 1 leaving all of the lanthanoids and Y in the solution. Fourth, ligands **4** and **5** are also selective precipitating agents for Sc and Th over other investigated REEs at pH 1, but a decrease in the U precipitation rate can be observed when compared to ligand **6**. Fifth, with ligand **4**, a dip in the precipitation percentages for most of the metals can be observed at pH 2.5, which is most likely resulting from the metal complexes dissolving back into the solution.

### Separation Factors

The ability to separate two elements from each other is expressed by separation factor (*SF*), which is calculated by adapting calculations from a liquid–liquid extraction as presented by Nelson et al.^48^ The ratio of precipitated metals and metals left in the solution on each pH is expressed as *D* (distribution factor) and can be determined according to the following eq 1, where [M]_p_ expresses the precipitated metals and [M]_s_ the metals in solution.
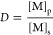
1

Separation factors between two elements can be determined with eq 2 by comparing their distribution factors.
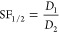
2

If metals precipitate completely from the solution or reversely, no precipitation occurs, it is not possible to determine the distribution factor and separation factor for the metal. Separation factors between adjacent lanthanoids, Sc, Th, and U were calculated for all of the ligands **1**–**6** (Tables S14–S19). Ionic radius (3+) of Y lies between the radii of Er and Tm, and therefore, Y was positioned between these two elements.

All ligands **16** have almost equal ability to separate adjacent lanthanoid pairs at each pH, meaning that no ligand was considerably better than the other. Additionally, separation factors between heavy adjacent lanthanoids from Er to Lu are calculated to be slightly better for all of the ligands **1**–**6** when compared to other adjacent lanthanoid pairs. For example, the best value for heavy lanthanoid pairs *SF*_Tm/Yb_ and *SF*_Yb/Lu_ arecalculated to be 4.33 ± 0.04 and 2.32 ± 0.02 with ligands **5** and **4**, respectively. These separation factors are higher than the reported separation factors for DEHPA and EHEHPA (*SF*_Tm/Yb_= 1.12–2.12, *SF*_Yb/Lu_ = 1.03–1.44) in the conventional liquid-liquid separation processes.^4^ For lighter lanthanoids separation factors are generally under two for the adjacent lanthanoids, with the exception of ligand **4** which has separation factor of 3.81 (±0.86) for Ce/La separation, which is in the same range with the conventional liquid-liquid method (*SF*_Ce/La_ 1.30–4.55), but shows improvement to the previously reported fractional crystallization with borates^15^ (*SF*_Ce/La_ 1.43) or oxalates^49^ (*SF*_Ce/La_ 1.5–2.5). With ligand **5**, albeit the obtained *SF*_Ce/La_ is lower, 2.11 ± 0.21, it is still in par with other separation systems reported above. For the other adjacent lanthanoids (Nd–Er), separation factors are calculated to be rather low for all of the ligands as they range from 1 to 1.7. When compared to other precipitation agents such as oxalates, the ligands **1**–**6** perform either similarly or slightly better, for example *SF*_Nd/Sm_ of 1.6 has been reported for oxalates,^49^ whereas for ligand **5**, a slightly better value is obtained (*SF*_Nd/Sm_ 2.0 ± 0.1). Compared to the hydrothermal borate crystallization, ligands **16** perform either similar or worse. However, a notable fact is that the borate crystallization requires high temperatures of 473 K for 3 days and additional 2 days for slow crystallization,^15^ whereas the precipitation of REEs and studied actinoids takes only 15 min at 295 K in acidic water solutions. As Y is positioned between Er and Tm, separation factors between these elements were also calculated. Good separation factors can be calculated for all of the ligands **16** for Tm/Y separation ranging from 2.06 to 8.88, of which the highest separation factor is obtained with ligand **1**. Smaller separation values (1.00–3.33) are obtained for Er/Y separation, the highest one (3.33 ± 1.17) is observed for ligand **3**.

Overall best separation factors are obtained when distribution factors of Sc, Th, or U are compared to the distribution factors of lanthanoids, for example, with ligand **5***SF*_Sc/La_ is calculated to be over 15 000 at pH 1 as Sc precipitates out from the solution almost quantitatively. In fact, with ligands **4–6**, *SF* cannot be calculated for Sc in most cases as it completely precipitates from the solution already at a low pH value (pH 1–2.5). Similar results are obtained for U and Th. For example, *SF*_Th/Lu_ is calculated to be 44.41 ± 6.34, 48.32 ± 6.04, and 33.62 ± 32.13 for ligands **4**–**6**, respectively, at low pH (2**–**2.5). These *SF*_Th/Lu_ are similar to *SF*_Th/Ln_ obtained from the fractional SeO_2_ crystallization in hydrothermal conditions.^19^ U precipitates completely from the solution with **6** at pH 1 and no separation factor can be calculated for U/Lu separation, whereas with **4** and **5**, the best separation factors are 9.45 ± 1.26 and 23.79 ± 0.24, respectively.

In general, ligands **1**–**6** provide improved separation factors in mild conditions especially for heavy adjacent lanthanoids and Y, when compared to oxalates or liquid–liquid extracting agents, and excellent selectivity toward Sc, Th, and U is observed with ligands **4**–**6**. Even though separation factor-wise the system is no better than the fractional crystallization with borates, the advantage is the simple and fast precipitation of the metals directly from the water solution at 295 K.

### Ligand and Metal Recovery

**Figure 4:**
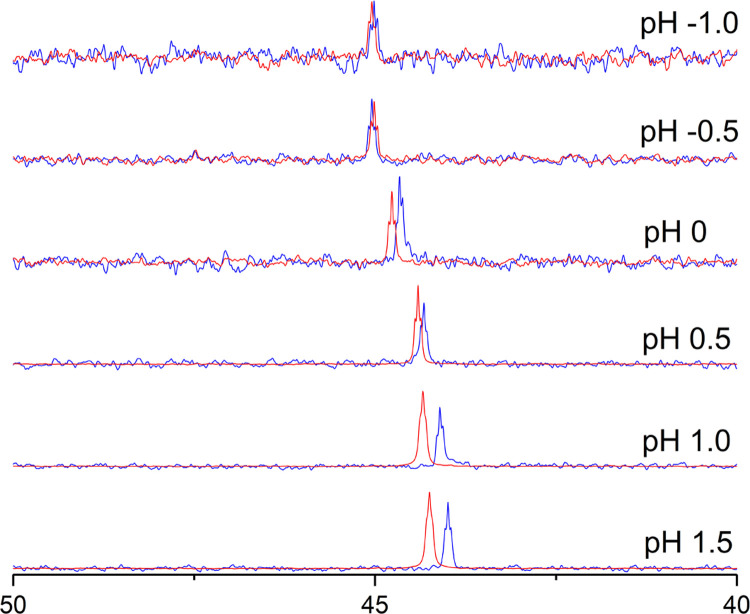
Three hundred megahertz ^31^P NMR spectra of ligand **1** Y complex (blue) and free ligand **1** (red) in different pH values.

Recovery of the ligands was investigated with the 1:3 complex of Y^3+^ and ligand **1** by measuring ^31^P NMR shifts of the complex in low pH values (1.5–(−1)) set with 65% HNO_3_, and comparing spectra to the NMR spectra of free ligand. It can be observed from Figure 4 that **1** is substituted for NO_3_^–^ around pH −0.5 because the ^31^P NMR shift of the Y-containing sample matches the shift of the free ligand **1** at this pH.

Importantly, no decomposition of the ligand can be observed in the ^1^H NMR spectrum of ligand during the recovery process. As the molarity of nitric acid in pH −0.5 can be calculated to be 3 molar, these findings not only show that the precipitated metals can be recovered from the complexes with 3 molar HNO_3_, but they also indicate that the investigated ligands **1**–**6** are recyclable and could be utilized more than once in the separation process.

## Conclusions

NMR and large-scale complexation and precipitation studies were performed for six different simple aminobis(phosphonates) ligands **1**–**6** with REEs, Th, and U. These studies were complemented by quantum chemical calculations and the acid–base titration in NMR scale to determine the protonation steps of the utilized ligands. The determined p*K*_a_ values of 1.3 and 5.6 for **1** suggested that **1**–**6** exist mainly as monoanionic ([**L**]^−^) form in the pH range used in the complexation and precipitation studies, whereas NMR titration studies in conjunction with computational data indicated that **1**–**6** preferably form either 1:1, 1:2, or 1:3 (metal–ligand) complexes in zwitterionic form with Lu, La, and Y, respectively. log*K* values for 1:1, 1:2, and 1:3 complexes, respectively, are calculated to be 2.4 ± 0.2, 4.9 ± 0.4, and 7.3 ± 0.3 for Y, 2.6 ± 0.5 and 4.4 ± 0.1 for La, and 2.1 ± 0.3 for Lu, in aqueous acidic solutions. Importantly, the precipitation studies showed that **4**–**6** are very selective precipitation agents to recover radioactive elements (Th and U) from REE concentrates in a short period of time (15 min). The performance of **1**–**6** to separate adjacent lanthanides was comparable or in some cases more efficient compared to other precipitation methods (borates and oxalates) reported so far. Additionally, the precipitation agents are recyclable in the separation process, as shown by the NMR study, and the metals could be recovered from the ligands by dissolving the formed complexes to 3 molar HNO_3_ without any decomposition of the ligands **1**–**6**. Considering all the abovementioned and the fact that aminobis(phosphonates) are relatively easy to synthesize with simple addition reaction, aminobis(phosphonates) are promising precipitation agents for REEs, Th, and U. Importantly, the selectivity of aminobis(phosphonates) toward adjacent lanthanoids could be increased by modification of ligand frameworks, which underpin their potential as alternative precipitation agents.

## Experimental Section

### Materials and Methods

Formaldehyde (36%) was purchased from VWR; phosphorous acid (99%) and hexylamine (98%) from Fluka Chemical Co.; 2-ethylhexylamine (98%), La(NO_3_)_3_·6H_2_O, and propylamine hydrochloride from Sigma-Aldrich; ethylamine hydrochloride (98%), butylamine (99%), and Y(NO_3_)_3_·6H_2_O (99.8%) from Merck; and Lu(NO_3_)_3_·H_2_O from abcr and amylamine (98%) from TCI chemicals. All of the chemicals were reagent grade and used without further purification. NMR measurements and titrations were performed on a Bruker Avance III 300 MHz-spectrometer, and NMR data was processed with Bruker TopSpin 4.0.8. IR spectra were measured by Bruker Alpha FT-IR. Elemental analyses were done by an Elementar Vario EL III-analysator. Lanthanoid concentrations were determined by a Perkin Elmer Optima 8300 DV ICP-OES- spectrometer.

### Syntheses

[(Ethylimino)bis(methylene)]bis(phosphonic acid) (**1**) was synthesized by dissolving phosphorous acid (19.35 g, 0.24 mol) and ethylamine (10.1 g, 0.05 mol) into a mixture of 100 mL of deionized water and 100 mL of 37% HCl. An excess of 36% formaldehyde (36 mL, 0.48 mol) was added dropwise to the solution for an hour, after which the solution was refluxed overnight at 120 °C. The solvent was removed under vacuum resulting in an oily product of which **1** was precipitated out with ethanol. The crude product was purified by recrystallization from hot ethanol to obtain it as a white solid. Yield 12.81 g, 46%. ^1^H NMR (D_2_O 300 MHz): δ 3.65–3.51 (m, 6H), and 1.39 (t, 3H). ^31^P NMR (D_2_O 300 MHz): δ 8.90. Elemental analysis Calcd for C_4_H_13_NO_6_P_2_: N, 6.01; C, 20.61; and H, 5.62. Found: C, 20.42; H, 5.68; and N, 5.92.

[(Propylimino)bis(methylene)]bis(phosphonic acid) (**2**) was prepared following the same procedure. The solvent was removed under vacuum resulting in a pale yellow oily product. A white precipitate was obtained after adding ethanol and heating up the solution. The crude product was purified by recrystallization from hot ethanol. Yield 7.27 g, 46%. ^1^H NMR (D_2_O 300 MHz δ): 3.60 (d, 4H), 3.51(m, 2H), 1.84 (m, 2H), and 1.02 (t, 3H). ^31^P NMR (D_2_O 300 MHz): δ 8.79. Elemental analysis calcd for C_5_H_15_NO_6_P_2_: C, 24.3; H, 6.12; and N, 5.67. Found: C, 24.3; H, 6.00; and N, 5.78.

[(Butylimino)bis(methylene)]bis(phosphonic acid) (**3**) was prepared following the same procedure. The solvent was removed under vacuum resulting in a yellow oily product. A white precipitate was obtained from adding ethanol and heating the solution. The crude product was purified by recrystallization from the water–ethanol solution. Yield 6.51 g, 41%. ^1^H NMR (D_2_O 300 MHz): δ 3.66–3.53 (m, 6H), 1.83 (m, 2H), 1.46 (m, 2H), and 1.01 (t, 3H). ^31^P NMR (D_2_O 300 MHz): δ 8.65. Elemental analysis calcd for C_6_H_17_NO_6_P_2_: C, 27.6; H, 6.56; and N, 5.36. Found: C, 27.1; H, 6.46; and N, 5.43.

[(Pentylimino)bis(methylene)]bis(phosphonic acid) (**4**) was prepared following the same procedure. The product precipitated out after cooling down. The white crude product was purified by recrystallization from water. Yield 5.83 g, 31%. ^1^H NMR (D_2_O 300 MHz): δ 3.66–3.52 (m, 6H), 1.85 (m, 2H), 1.41 (m, 4H), and 0.96 (t, 3H). ^31^P NMR (D_2_O 300 MHz): δ 8.56. Elemental analysis calcd for C_7_H_19_NO_6_P_2_: C, 30.55; H, 6.96; and N, 5.09. Found: C, 29.74; H, 6.91; and N, 5.02.

[(Hexylimino)bis(methylene)]bis(phosphonic acid) (**5**) was prepared following the same procedure. Around 1 h, after starting the refluxing, brown solid started forming into the solution. After cooling down and stirring the solution for ∼15 min, white solid precipitated heavily out, and it was isolated by suction filtration. The crude product contained still some brown impurities, which were removed by dissolving the product in hot water and filtrating while hot. The crude product was purified by recrystallization from hot water, and colorless needles were obtained. Yield 6.36 g, 36%. ^1^H NMR (D_2_O 300 MHz): δ 3.71–3.50 (m, 6H), 1.84 (m, 2H), 1.52–1.29 (m, 6H), and 0.93 (t, 3H). ^31^P NMR (D_2_O 300 MHz): δ 8.66. Elemental analysis calcd for C_8_H_21_NO_6_P_2_: C, 33.22; H, 7.32; and N, 4.84. Found: C, 32.79; H, 7.23; and N, 4.82.

[(2-Ethylhexylimino)bis(methylene)]bis(phosphonic acid) (**6**) was prepared by refluxing the reaction mixture for 3 h instead of 12 h at 120 °C. The solution was concentrated and the left stand at the room temperature overnight. The precipitated white solid was filtrated, washed with cold water, and purified by recrystallization from hot water. Yield 11.46 g, 46%. ^1^H NMR (D_2_O 300 MHz): δ 3.61 (d, 4H), 3.54 (m, 2H), 1.98 (m, 1H), 1.59–1.29 (m, 8H), and 0.95 (m, 6H). ^31^P NMR (D_2_O 300 MHz): δ 8.37. Elemental analysis calc. (%): N: 4.42, C: 37.86, and H: 7.94; meas. (%): N: 4.173, C: 37.12, and H: 7.972. Elemental analysis calcd for C_10_H_25_NO_6_P_2_: C, 37.86; H, 7.94; and N, 4.42. Found: C, 37.12; H, 7.97; and N, 4.17.

### Deprotonation Titration

Three hundred milligrams of **1** was dissolved into 9 mL of D_2_O to obtain a 0.14 M solution. Nondeuterated 5% NH_3_ solution was added to the stock solution, and from each 0.5 pH, the NMR sample was taken aside. The pH was measured in the range of 0.5 to 10.5.

### NMR Titrations

Titrating **1** with Y(NO_3_)_3_: 0.01 M solution of ligand **1** was prepared by dissolving ligand **1** (10.249 mg, 0.044 mmol) into 4.4 mL of D_2_O. Typically, 0.6 mL of analyte was taken aside, and roughly 20 times excess of Y(NO_3_)_3_·6 H_2_O (303.07 mg, 0.791 mmol) was added to the titrant. The analyte was titrated by adding 0.1 equiv of the titrant (4 μL) to the analyte, and ^31^P NMR spectra was measured after each addition. The analyte was titrated until the concentration reached 1 equiv. Titrations were performed similarly with La(NO_3_)_3_ and Lu(NO_3_)_3_ by preparing 0.01 M solution of **1** into 3 mL of D_2_O, taking 0.6 mL analyte aside and adding excess La(NO_3_)_3_ (88.00 mg, 0.203 mmol) or Lu(NO_3_)_3_ (61.64 mg, 0.17 mmol) into the titrant. Analyte was titrated by adding 0.1 equiv (10 μL) to the titrant until 1 equiv was reached. pH for the Lu titration was set to 1.0 to prevent the complex from precipitating. All titrations were replicated three times.

Titrating Y(NO_3_)_3_, La(NO_3_)_3_ with **1**: Titrations were done by following the same procedure. The analyte was titrated by adding 0.3 equiv of the titrant (7 μL) to the analyte until the concentration reached 5 equiv.

All titrations were replicated three times and pH was monitored during titrations. Binding models were fitted with HypNMR2008 programme Version 4.0.71.^50^

### Precipitation Experiments

Two hundred fifty milligrams of ligands **1**–**6** were dissolved into 100 mL of 5% HNO_3_ prepared from ultrapure water to avoid any unwanted element contaminations. Typically, 1 g/L uranium standard solution was diluted (10/100 mL 5% HNO_3_) to obtain 100 mg/L solution, and 1.7 mL of the solution was combined with 17.3 mL of the 10 mg/L REE multistandard solution (Ln, Sc, Y, Th) to obtain roughly 9 mg/L solution for the inspected metals. For each of the ligands **1**–**6**, 3 mL of the metal solution and 3 mL of the ligand solution were combined and pH was set with 5% NH_3_, prepared in ultrapure water. From each pH increment of 0.5 in the pH range of 1–4, and before adding ammonia (pH 0), 0.5 mL of the sample was taken aside, filtrated with syringe, and diluted to 5 mL with 5% HNO_3_ for the ICP-OES measurements. The measurements were replicated three times. Precipitation experiments were also performed for the solutions without ligands **1**–**6**, to investigate the precipitation of metals in the absence of ligands.

### Ligand Recovery

Roughly 0.01 M solution of the 1:3 metal–ligand complex of Y with**1** was prepared by dissolving 16.45 mg of **1** and 9.07 mg of Y(NO_3_)_3_ into 6 mL of D_2_O. The pH of the solution was set with 65% HNO_3_, and samples were taken from the solution every 0.5 change in pH within the pH range of 1.5 to −1. For comparision, roughly 0.01 M solution of free ligand **1** was prepared by dissolving 4.97 mg of **1** into the 1 −2 mL of D_2_O. pH was set similarly with 65% HNO_3_ and samples were taken aside every 0.5 pH. ^31^P NMR spectra of each sample were measured.

### Computational Details

The lowest energy structure for the 1:3 complex of Y^3+^ and three [L**1**]^−^ in the neutral and zwitterionic form was obtained from the conformational sampling, which were followed by the three different separate DFT calculations. The conformational sampling was carried out employing the Merck Molecular Force Field (MMFF)^51^ with Monte-Carlo search as implemented in Spartan’ 18 molecular modeling software.^52^ The same software was also used in the subsequent PBE-D3/def2-SV(P)^53−59^ single-point energy calculations that were carried out for all 1483 and 901 unique structures of neutral and zwitterionic forms, respectively, obtained from the conformational sampling. Out of these structures, 277 (198) lowest energy structures of the neutral (zwitterionic) form were selected to the full geometry optimizations performed at the PBE1PBE-D3/def2-SV(P)^57−61^ level of theory in the gas-phase because no clear energy cut-off value could be determined from the results of the single-point energy calculations. These calculations were carried out with Gaussian 16 quantum chemistry program.^62^ For both forms, the subsequent final geometry optimizations were performed for the 10 lowest energy structures obtained from the previous step at the PBE1PBE-D3/def2-TZVP^57−61^ level in a solution state. In the solution-state calculations, water was used as a solvent and it was modeled using the integral equation formalism variant of the polarizable continuum model as implement in the Gaussian 16.^63−66^ The frequency analyses were calculated for all of the final optimized structures to ensure that they correspond to a true minimum on the potential energy hypersurface (no negative frequencies).

## Supporting Information Available

Characterization for the aminobis(phosphonates) **1–6** (^1^H NMR and IR spectra) NMR titration curve for ligand **1**, spectra, and plotted shifts for the complexation studies; Tables for the separation factors between adjacent lanthanoids and ^31^P NMR spectra for the ligand recovery (PDF)
