# Supplementary material for: Alkyl-Substituted Aminobis(phosphonates)—Efficient Precipitating Agents for Rare Earth Elements, Thorium, and Uranium in Aqueous Solutions
Source: ACS Omega. 2021 Sep 13;6(37):23977–87. doi: 10.1021/acsomega.1c02982 (PMC8459412; doi:10.1021/acsomega.1c02982)
Supplement: Supplementary file 1 — ao1c02982_si_001.pdf [file ao1c02982_si_001.pdf]

Supporting Information

Alkyl substituted aminobis(phosphonates) – efficient precipitating agents for rare earth elements, thorium and uranium in aqueous solutions.

Emilia J. Virtanen,[a] Siiri Perämäki,[b] Kaisa Helttunen,[a] Ari Väisänen*,[b] Jani O. Moilanen*[a]

a) Department of Chemistry, Nanoscience Center, University of Jyväskylä. P.O. Box 35, FI-40014 Jyväskylä, Finland

b) Department of Chemistry, University of Jyväskylä. P.O. Box 35, FI-40014 Jyväskylä, Finland

Contents

Aminobis(phosphonate) 1-6 characterization: ^{1}H NMR and IR-spectra S3

Water solubilities S9

^{1}H and ^{31}P NMR titration curve S9

^{1}H NMR spectra for complexation titration S10

^{31}P NMR spectra for metal-to-ligand complexation titration S11

Metal-to-ligand binding model fits for Y, La and Lu S12

^{31}P NMR spectra for ligand-to-metal complexation titration S13

Ligand-to-metal binding model fits for Y, La and Lu S14

F-tests for binding models S15

Computed structures S17

Precipitation percentages for ammonia S18

Precipitation percentages and errors for precipitation with ligands 1-6 .... S19

Separation factors for adjacent lanthanoids S25

Computational data S28

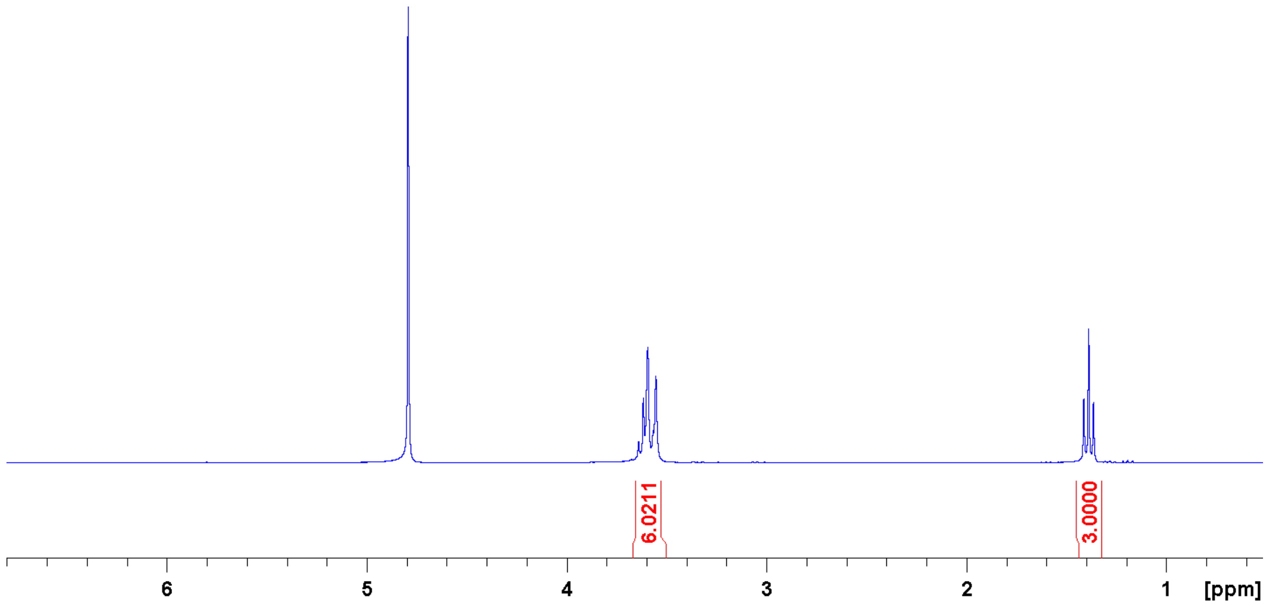

Figure S1. ^{1}H NMR 300 MHz spectrum for ligand **1** measured in D_2O

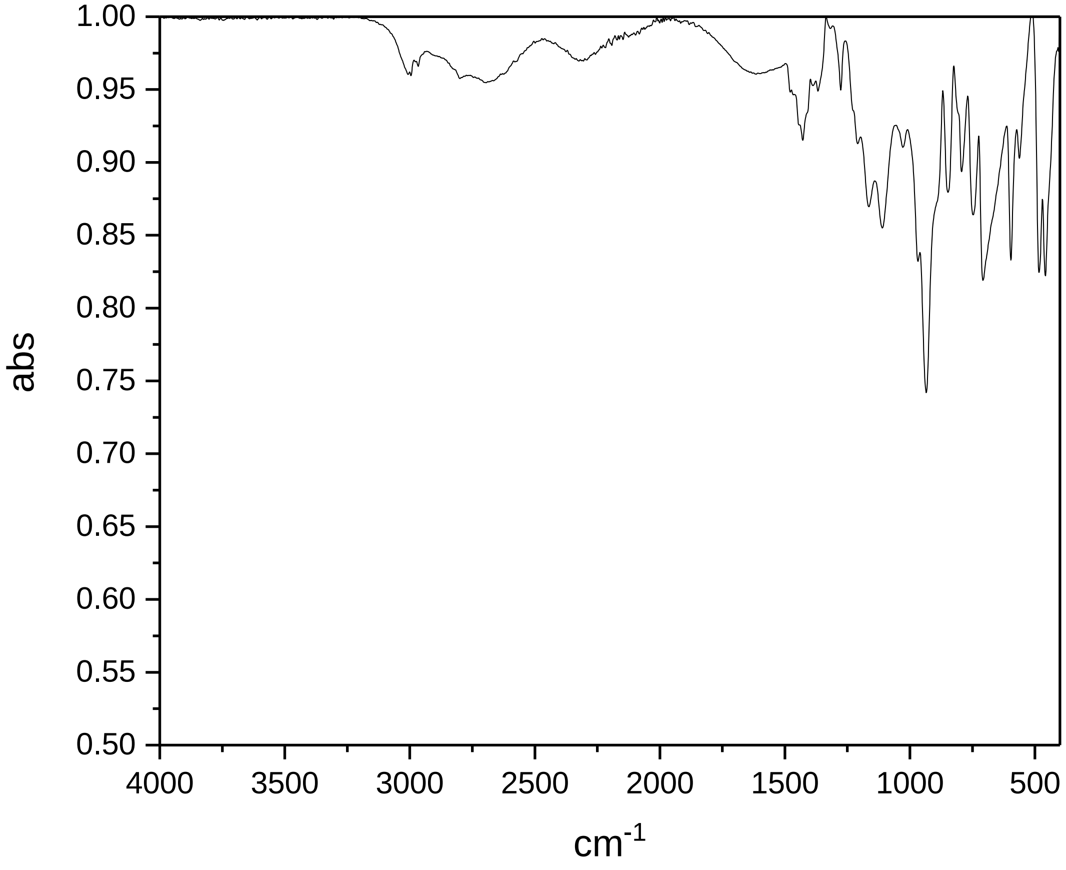

Figure S2. FT-IR spectrum for ligand **1**

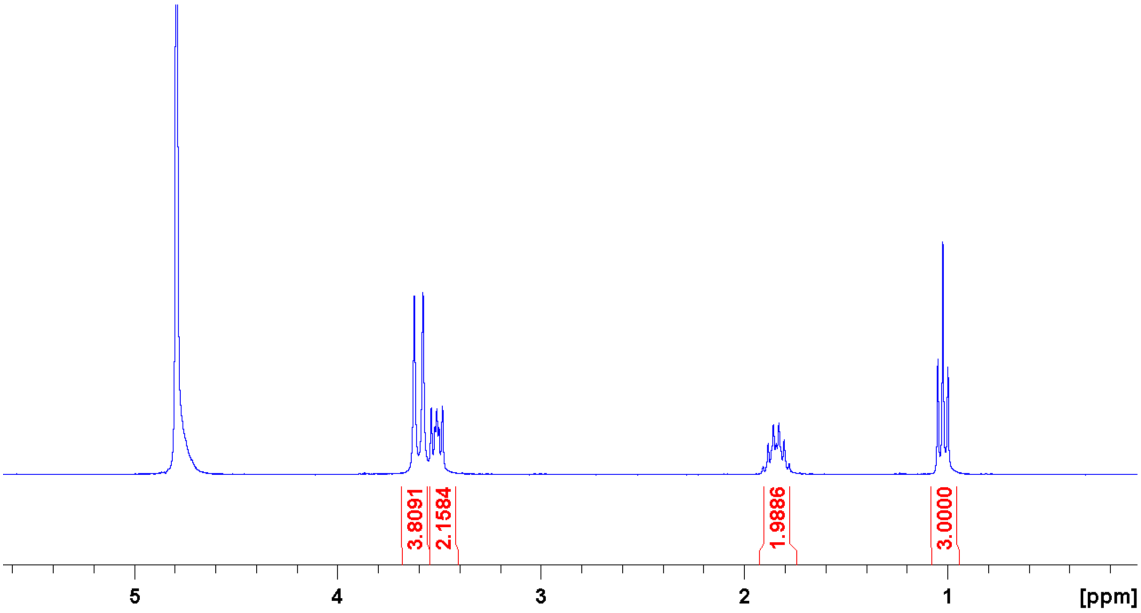

Figure S3. ^{1}H NMR 300 MHz spectrum for ligand **2** measured in D_2O

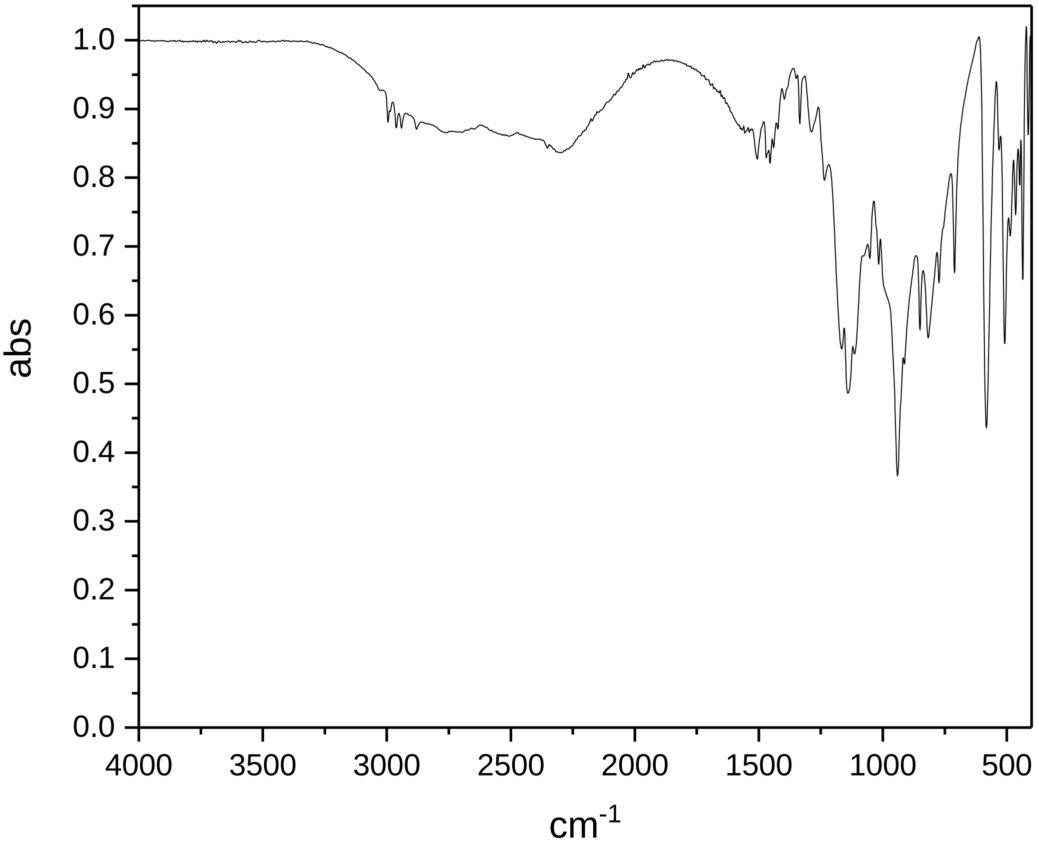

Figure S4. FT-IR spectrum for ligand **2**

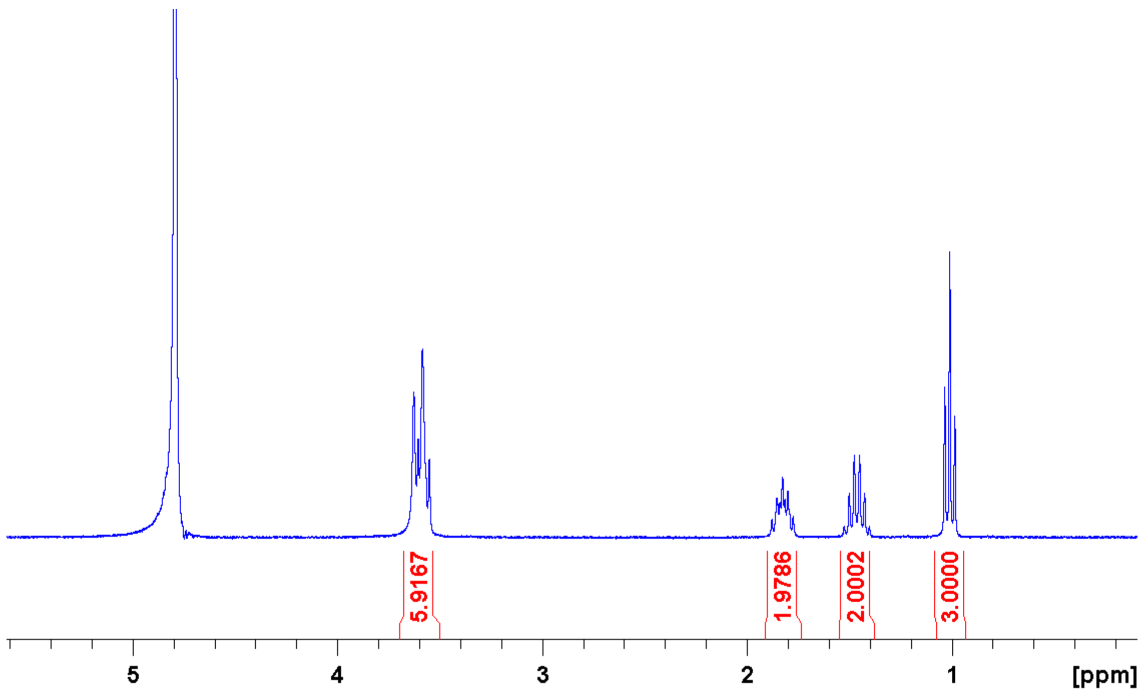

Figure S5. ^{1}H NMR 300 MHz spectrum for ligand **3** measured in D_2O

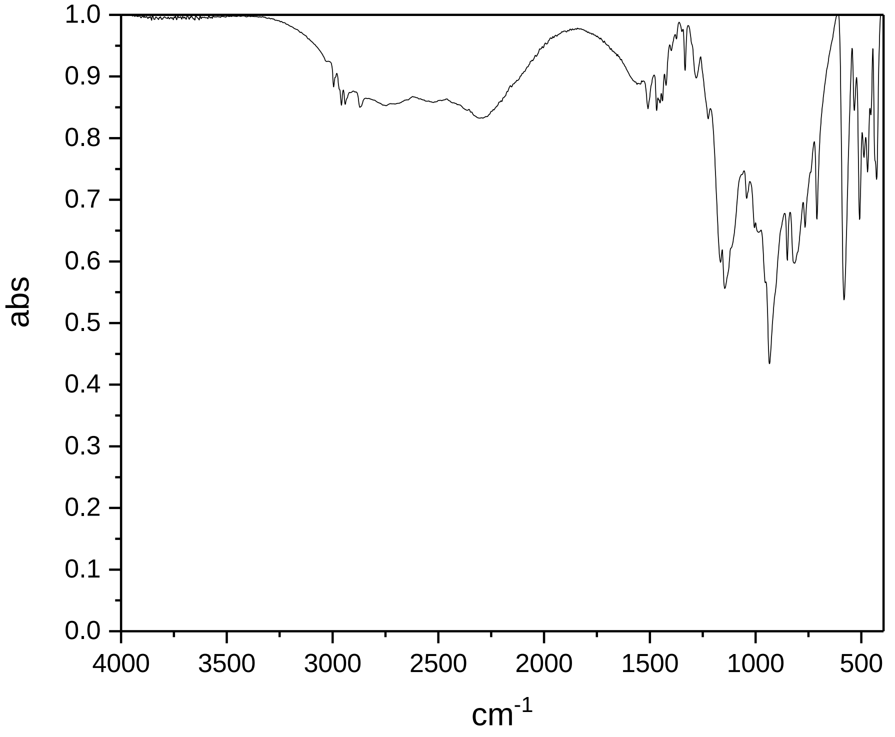

Figure S6. FT-IR spectrum for ligand **3**

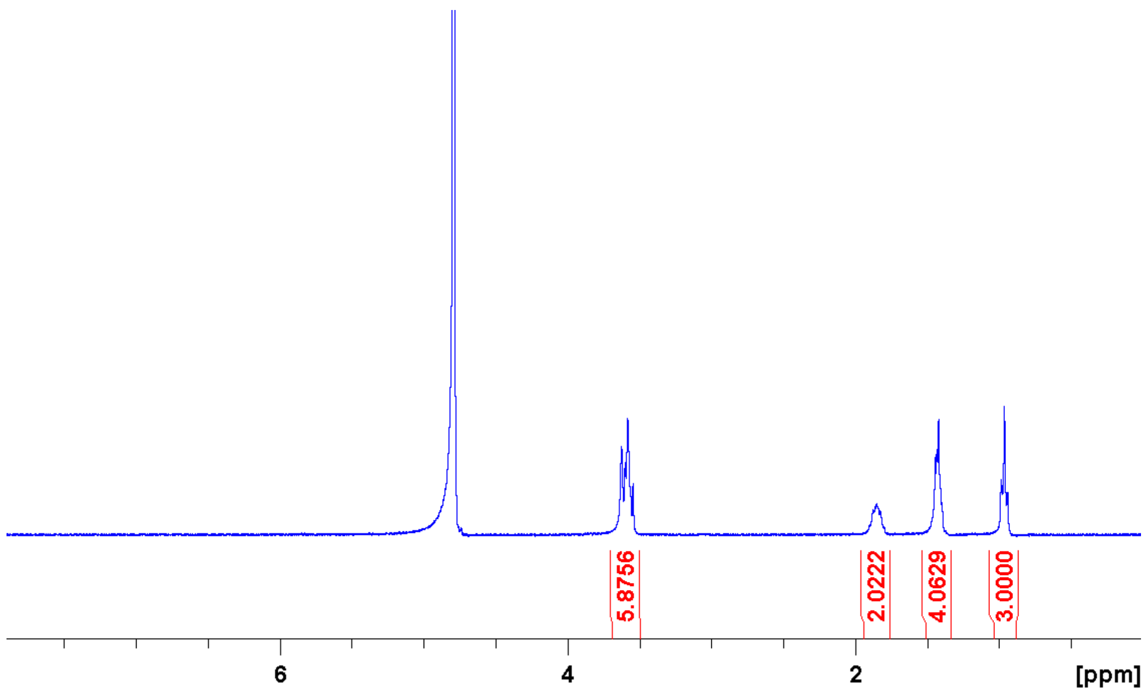

Figure S7. ^{1}H NMR 300 MHz spectrum for ligand **4** measured in D_2O

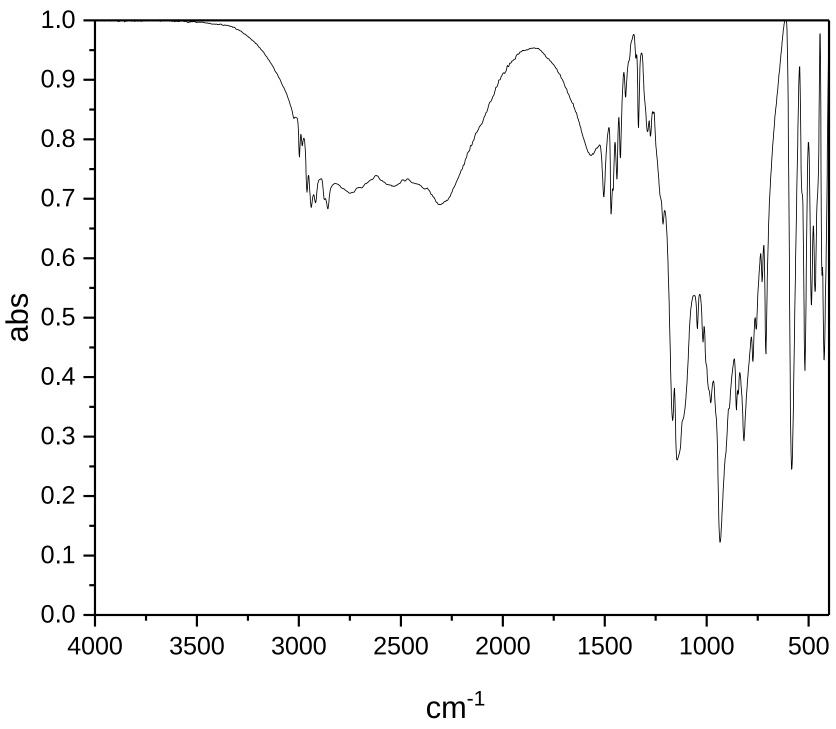

Figure S8. FT-IR spectrum for ligand **4**

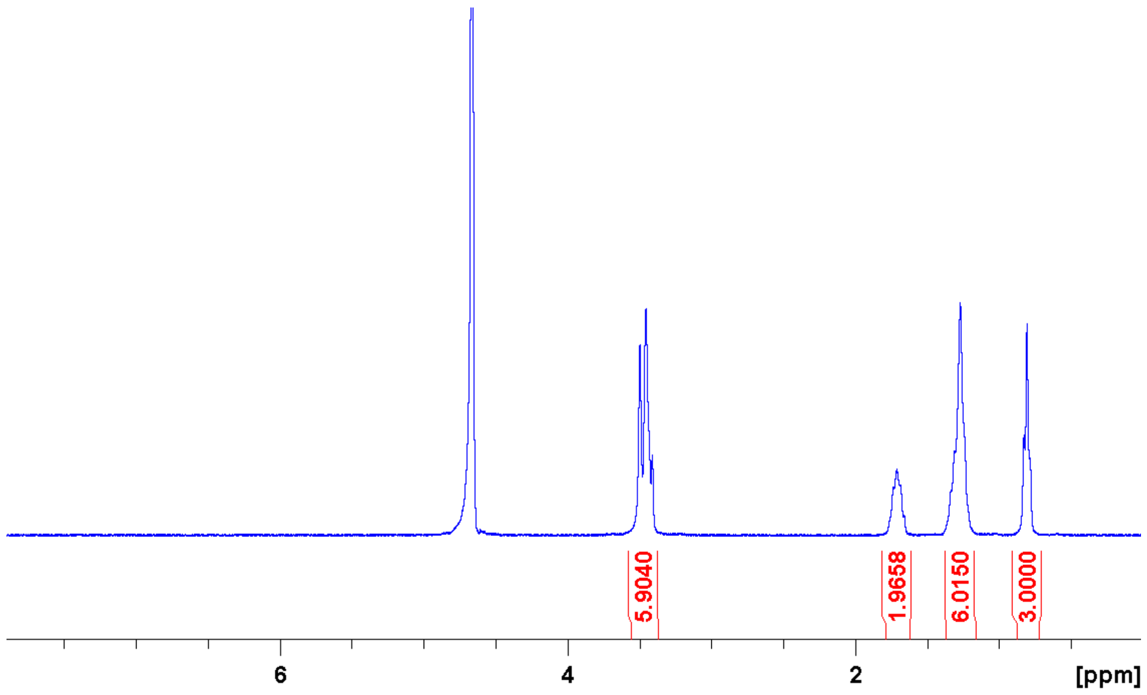

Figure S9. ^{1}H NMR 300 MHz spectrum for ligand **5** measured in D_2O

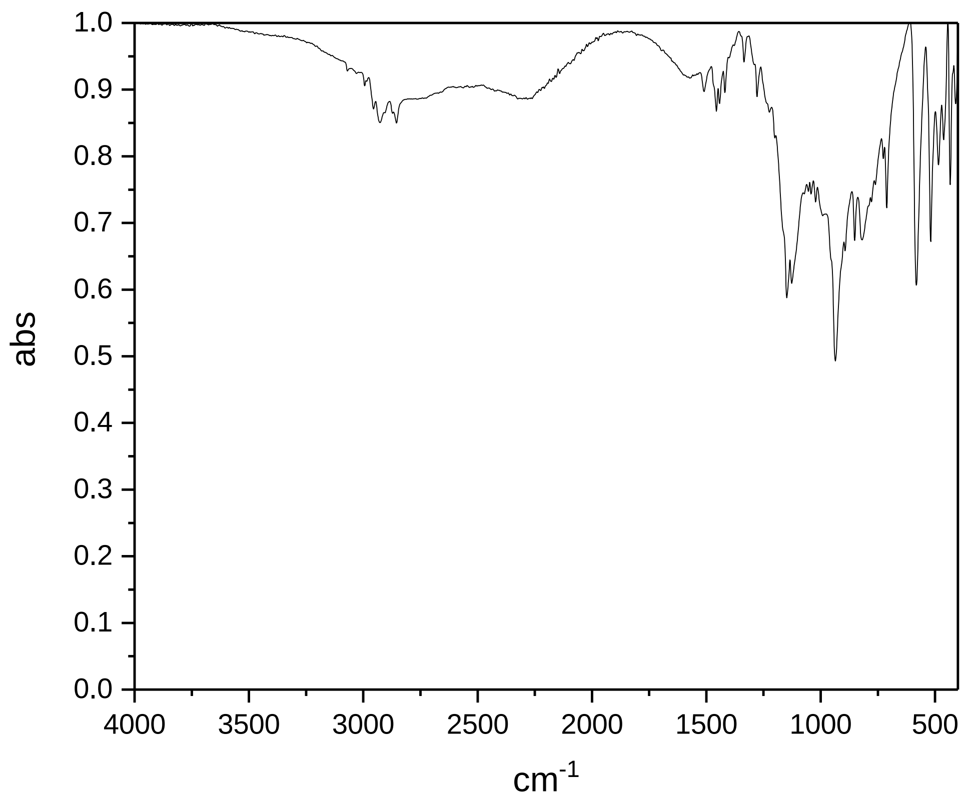

Figure S10. FT-IR spectrum for ligand **5**

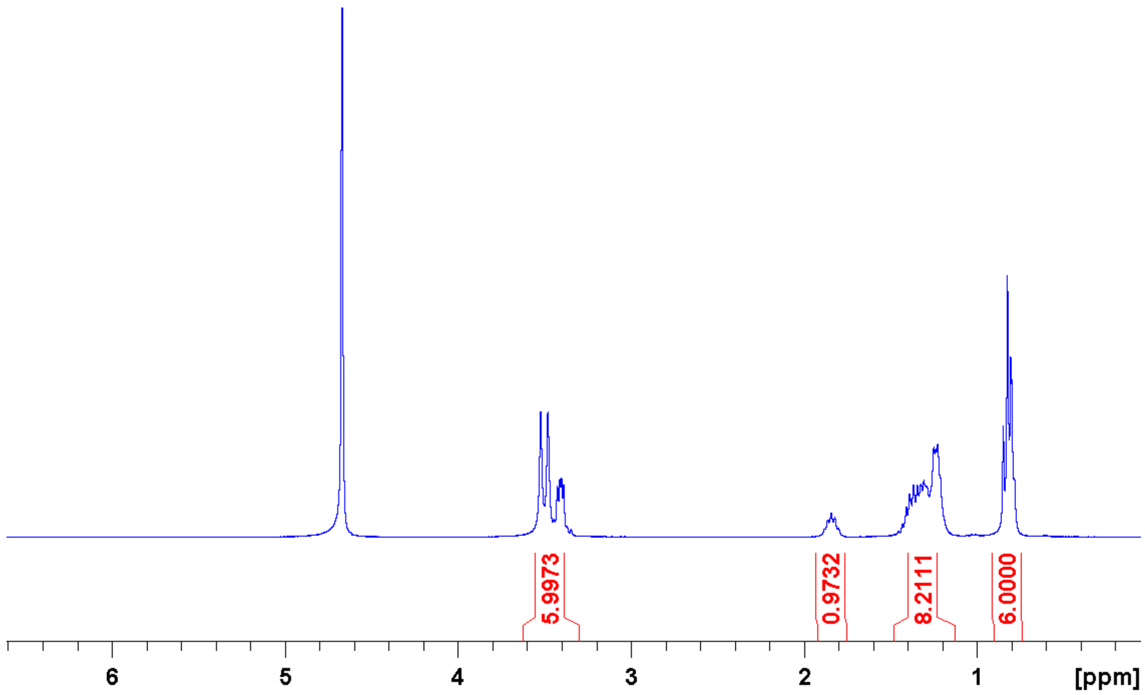

Figure S11. ^{1}H NMR 300 MHz spectrum for ligand **6** measured in D_2O

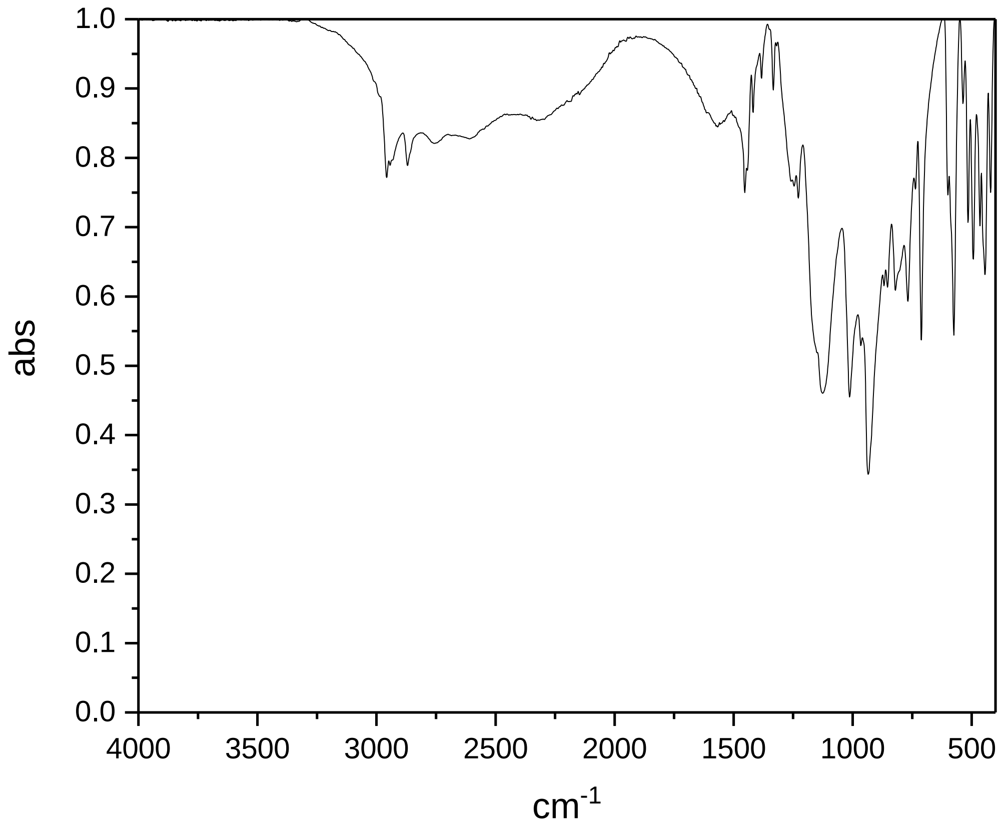

Figure S12. FT-IR spectrum for ligand **6**

Table S1. Calculated water solubilities for ligands **1-6**. Water solubility of the ligands were determined by dissolving compounds into water until saturated solution was obtained. Solutions were then filtrated, left to evaporate and residues were weighted where the water solubilities were calculated for each ligand.

Ligand	1	2	3	4	5	6
**m (g)**	1.470	1.5316	0.8314	1.1474	0.105	0.079
**V (ml)**	4.5	5	4.5	10	5	8.3
**solubility (g/l)**	326.7	306.32	184.8	114.7	21 g/l	9.5

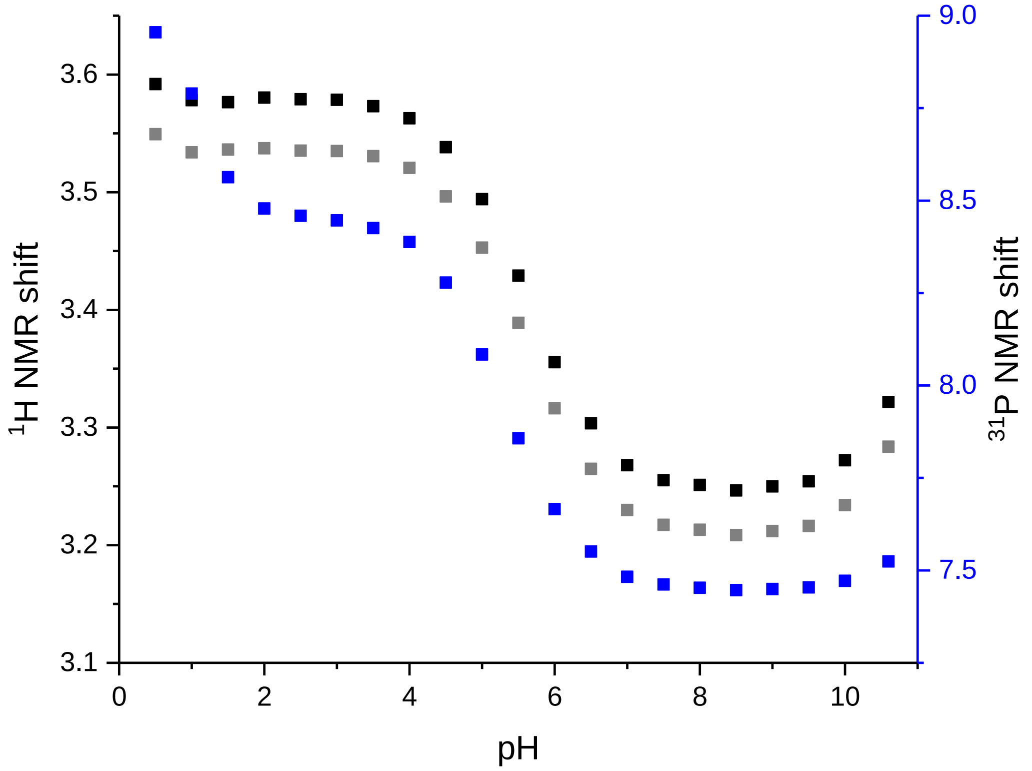

Figure S13. Measured ^{1}H NMR (black and grey) and ^{31}P NMR (blue) shifts for **1** in D_2O in the pH range of 0.5 – 10.5.

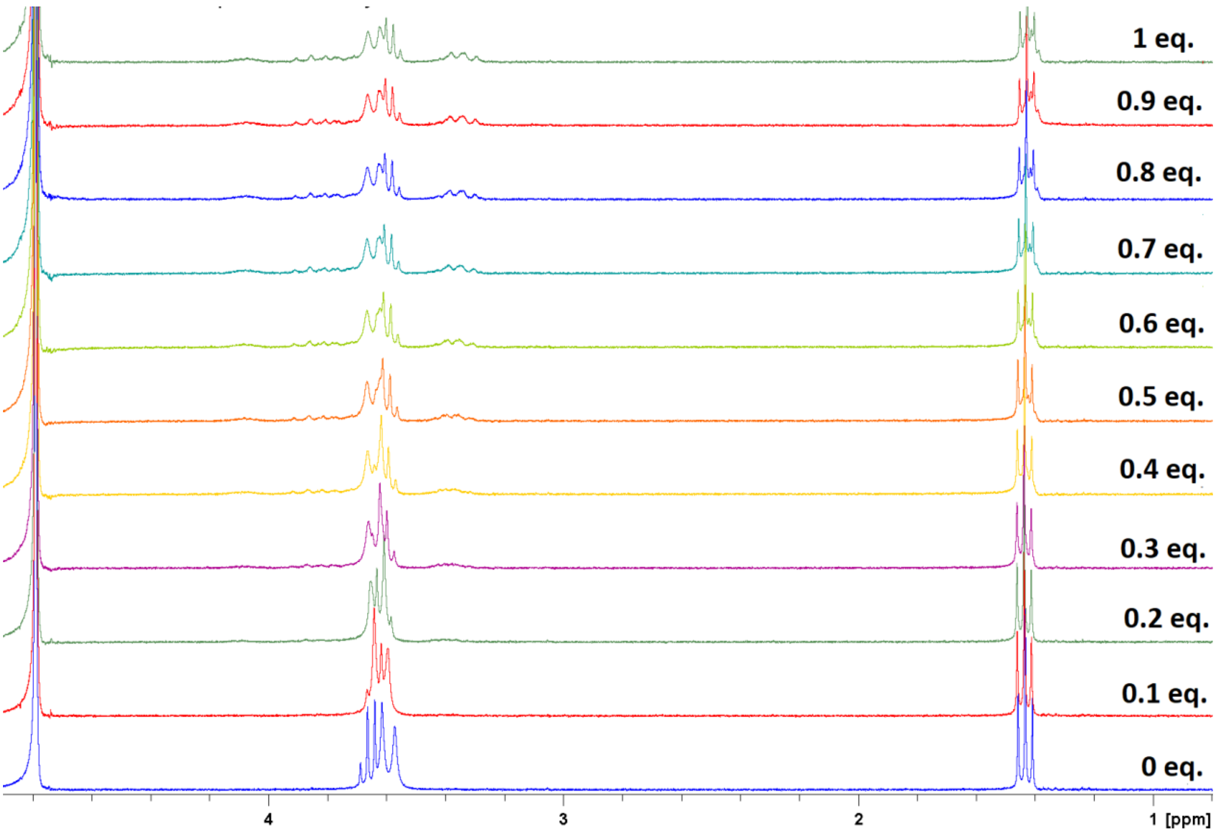

Figure S14. 300 MHz ^{1}H NMR shifts in D_2O when ligand **1** is titrated with $Y(NO_3)_3$

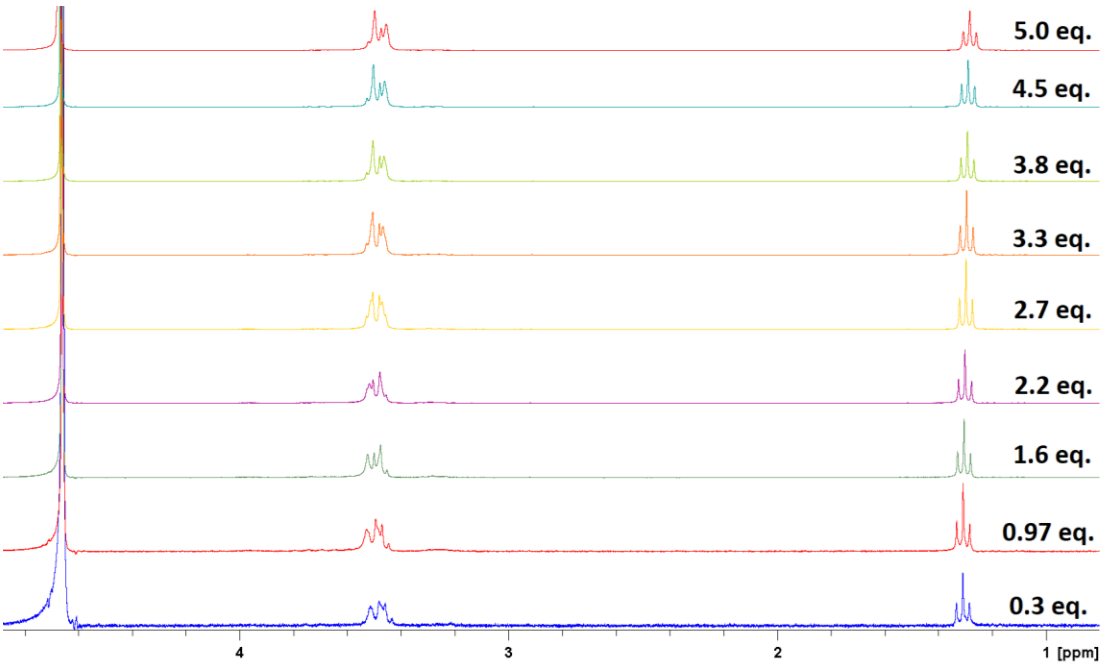

Figure S15. 300 MHz ^{1}H NMR shifts in D_2O when $Y(NO_3)_3$ is titrated with ligand **1** by intervals of roughly 0.6 equivalent of added ligand.

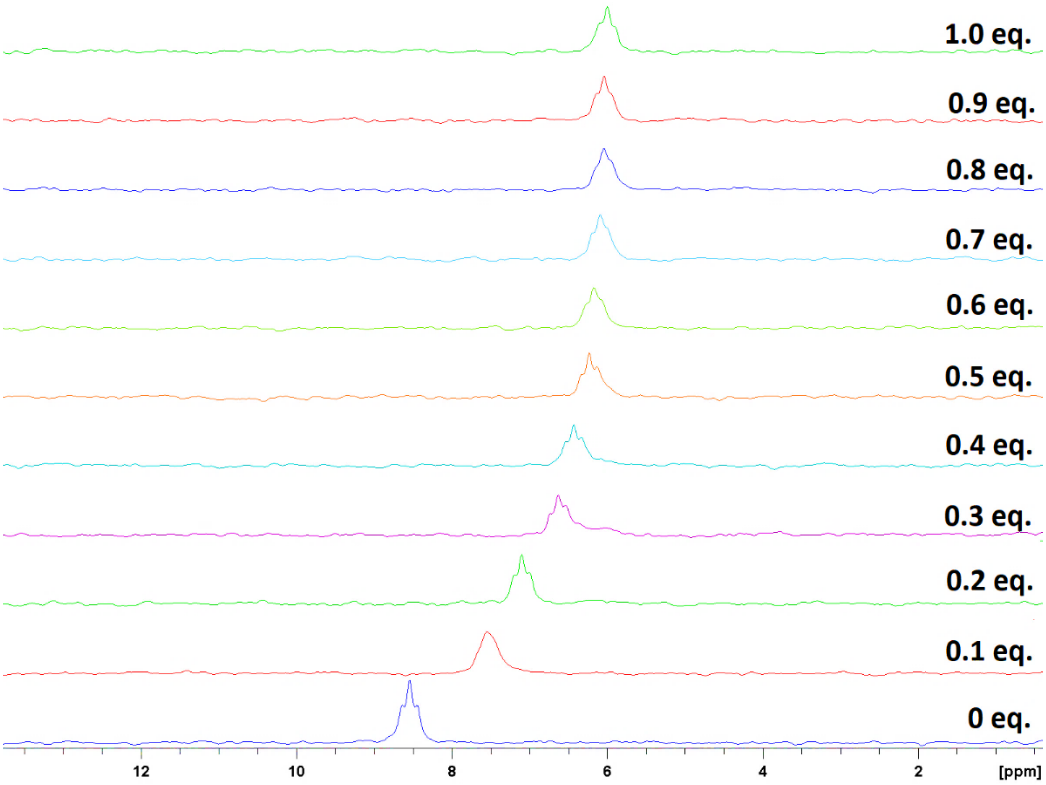

Figure S16. 300 MHz ^{31}P NMR shifts in D_2O when ligand **1** is titrated with $Y(NO_3)_3$

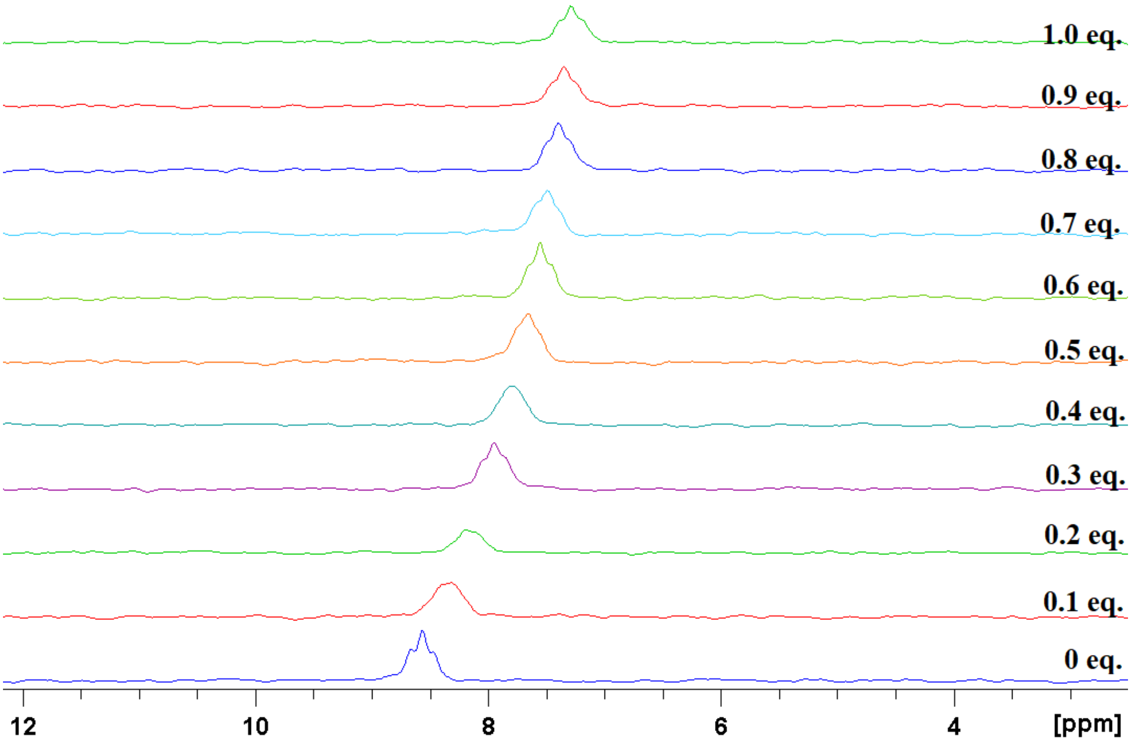

Figure S17. 300 MHz ^{31}P NMR shifts in D_2O when ligand **1** is titrated with $La(NO_3)_3$

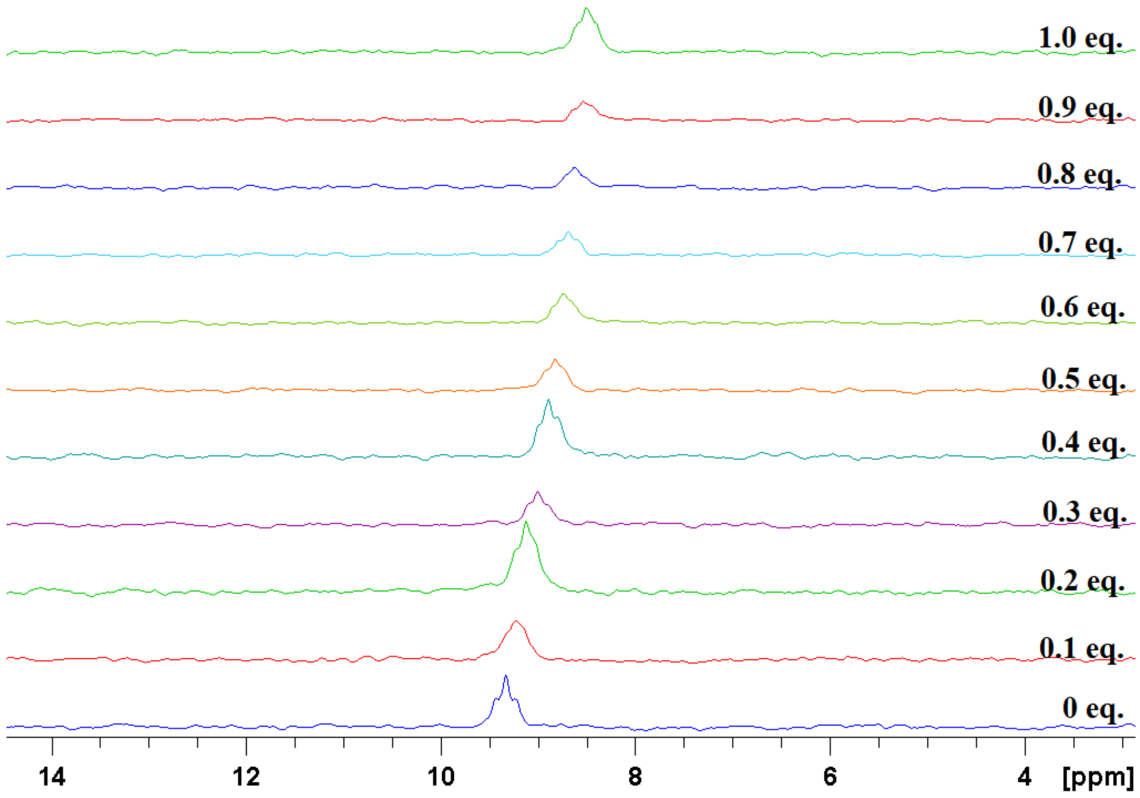

Figure S18. 300 MHz ^{31}P NMR shifts in D_2O when ligand **1** is titrated with $Lu(NO_3)_3$

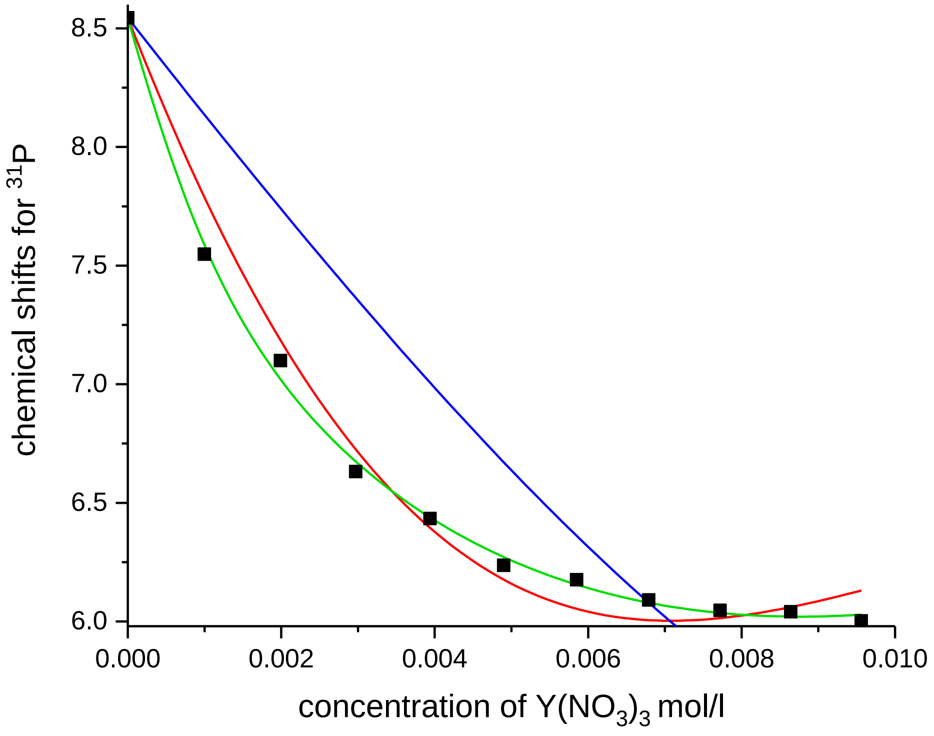

Figure S19. ^{31}P NMR shift change as the function of $Y(NO_3)_3$ concentration as well as fittings for 1:1 (blue), 1:2 (red) and 1:4 (green) M:L binding models when **1** is titrated with $Y(NO_3)_3$.

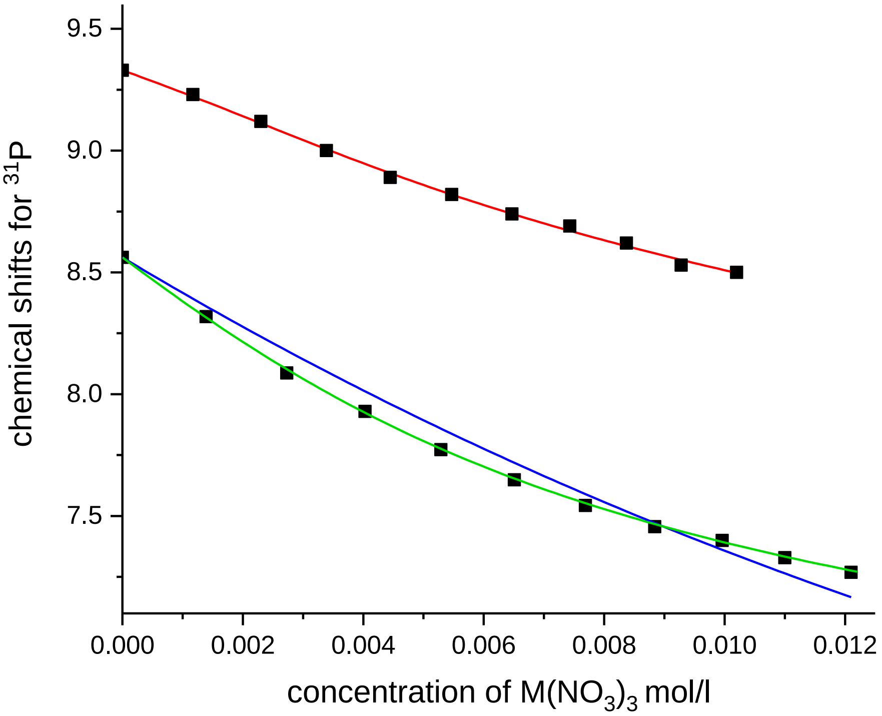

Figure S20. ^{31}P NMR shift change as the function of $M(NO_3)_3$ concentration (M = La or Lu) as well as fittings for M:L binding models for La (1:1 (blue) and 1:3 (green)) and for Lu: (1:2 (red)) when **1** is titrated with $M(NO_3)_3$.

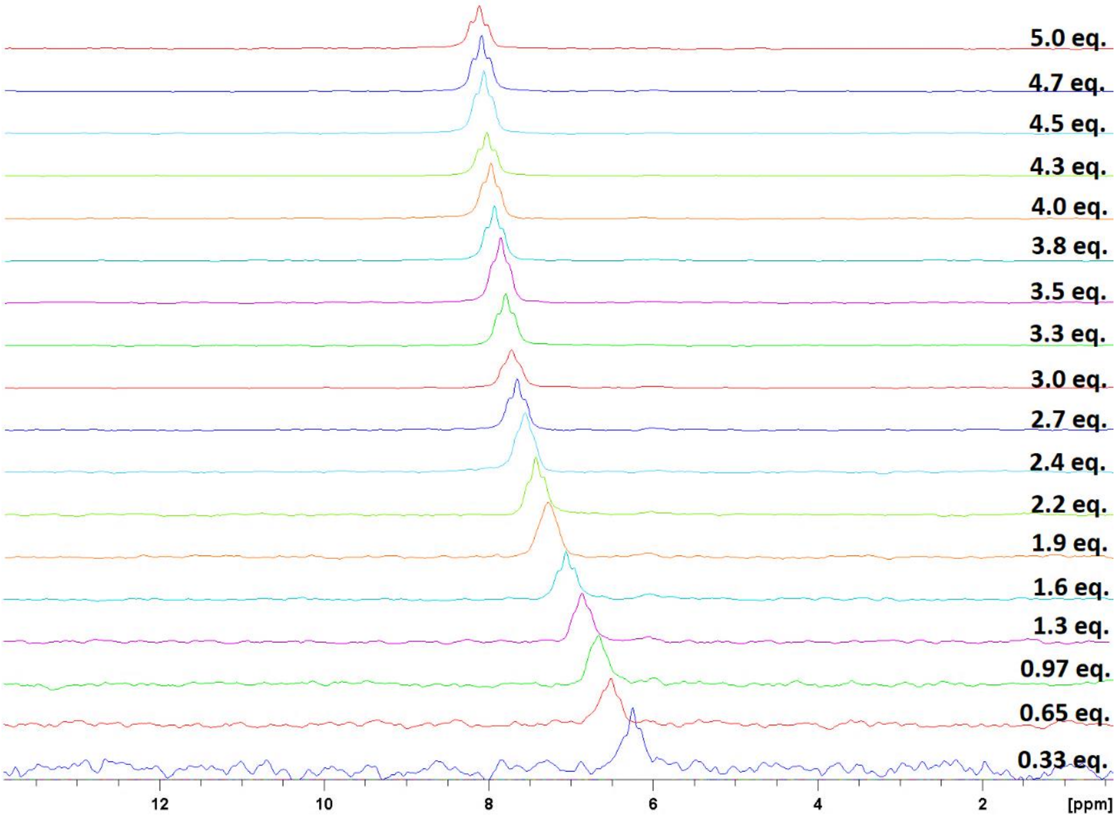

Figure S21. 300 MHz ^{31}P NMR shifts in D_2O when $Y(NO_3)_3$ is titrated with ligand **1**.

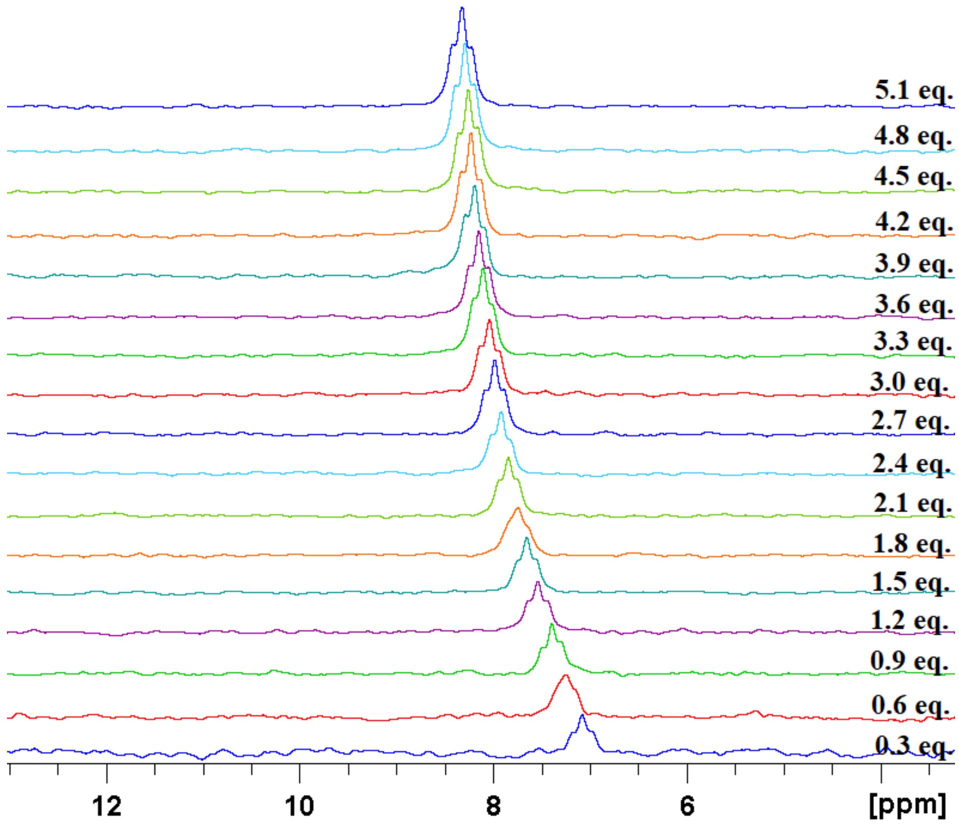

Figure S22. 300 MHz ^{31}P NMR shifts in D_2O when $La(NO_3)_3$ is titrated with ligand **1**.

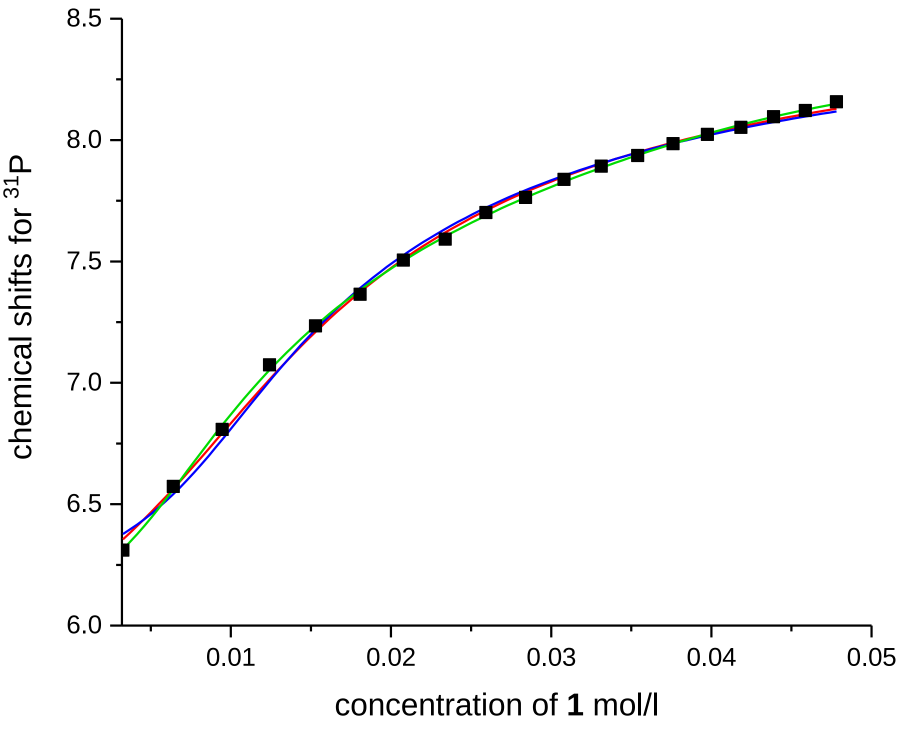

Figure S23. ^{31}P NMR shift change as the function of the ligand **1** concentration, and fittings for 1:1 (blue), 1:2 (red) and 1:4 (green) M:L binding models when $Y(NO_3)_3$ is titrated with **1**.

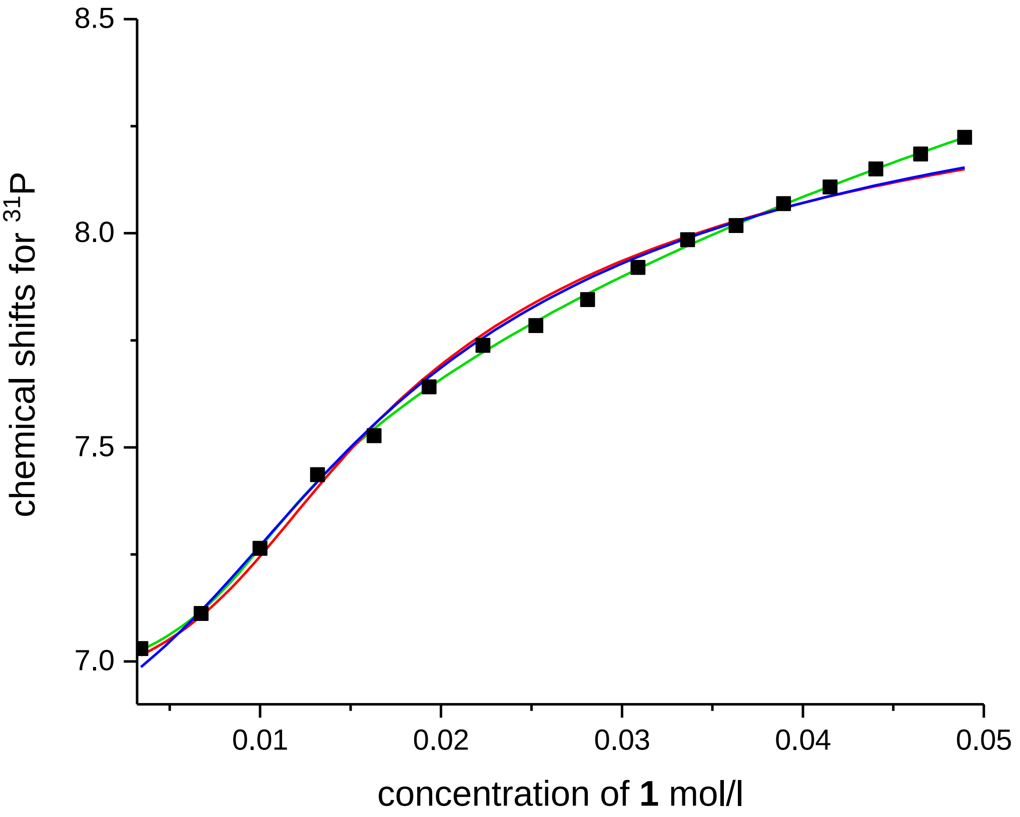

Figure S24. ^{31}P NMR shift change in the function of the ligand **1** concentration, and fittings for 1:1 (blue), 1:2 (red) and 1:4 (green) M:L binding models when $La(NO_3)_3$ is titrated with **1**.

Table S2. F-tests for 1:3 and 1:4 binding models when **1** is titrated with $Y(NO_3)_3$. P-value > 0.05 indicates that 1:3 model fits significantly better than 1:4 model.

		**Sum of squares**		**Degrees of freedom**
Dataset	**Repeat 1**	**Repeat 2**	**Repeat 3**	
1:3 model	1.377×10^{-2}	2.714×10^{-4}	1.501×10^{-3}	6
1:4 model	1.256×10^{-2}	3.726×10^{-4}	8.882×10^{-4}	4
Difference	1.203×10^{-3}	-1.002×10^{-4}	6.129×10^{-4}	2
Relative difference	0.0958	-0.2696	0.690	0.5
Ratio (F)	0.1915	-0.5392	1.380	
Numerator	2	2	2	
Denominator	4	4	4	
P-value	0.8329	n/a[1]	0.3501	

[1]P-value couldn’t be determined as ratio (F) is negative.

Table S3. F-tests for 1:2 and 1:3 binding models when **1** is titrated with $La(NO_3)_3$. For repeat 3, adding third binding constant worsened the fit, therefore F-test was not conducted. P-value > 0.05 indicates that 1:2 model fits better.

	Sum of squares		Degrees of freedom	
Dataset	**Repeat 1**	**Repeat 2**	**Repeat 1**	**Repeat 2**
1:2 model	3.596×10^{-3}	1.849×10^{-3}	4	4
1:3 model	0.388×10^{-3}	1.793×10^{-3}	6	6
Difference	3.207×10^{-3}	0.559×10^{-4}	2	2
Relative difference	8.258	0.0312	1	0.4
Ratio (F)	8.258	0.0781		
Numerator	2	2		
Denominator	2	5		
P-value	0.1080	0.9261		

Table S4. F-tests for 1:1 and 1:2 binding models when **1** is titrated with $Lu(NO_3)_3$. P-value > 0.05 indicates that 1:1 model fits better.

		Sum of squares		Degrees of freedom
Dataset	**Repeat 1**	**Repeat 2**	**Repeat 3**	
1:1 model	0.779×10^{-3}	0.429×10^{-3}	0.195×10^{-2}	2
1:2 model	1.793×10^{-3}	0.380×10^{-3}	1.434×10^{-3}	4
Difference	1.014×10^{-3}	0.488×10^{-4}	0.516×10^{-3}	2
Relative difference	0.5656	0.1284	0.3598	0.28571
Ratio (F)	1.9795	0.4493	1.2591	
Numerator	2	2	2	
Denominator	7	7	7	
P-value	0.2083	0.6553	0.3411	

Table S5. F-tests for 1:3 and 1:4 binding models when $Y(NO_3)_3$ is titrated with **1**. P-value > 0.05 indicates that 1:3 model fits better for repeats 2 and 3.

		Sum of squares		Degrees of freedom
Dataset	**Repeat 1**	**Repeat 2**	**Repeat 3**	
1:3 model	2.392×10^{-2}	1.718×10^{-3}	2.530×10^{-3}	13
1:4 model	1.077×10^{-2}	1.715×10^{-3}	2.341×10^{-3}	11
Difference	1.315×10^{-2}	2.88×10^{-6}	0.189×10^{-3}	2
Relative difference	1.221	1.679×10^{-3}	8.074×10^{-2}	0.181818
Ratio (F)	6.716	9.236×10^{-3}	0.4037	
Numerator	2	2	2	
Denominator	11	11	11	
P-value	0.0124	0.9909	0.6783	

Table S6. F-tests for 1:3 and 1:4 binding models when $La(NO_3)_3$ is titrated with **1**. P-value > 0.05 indicates that 1:3 model fits better for repeats 1 and 3.

		**Sum of squares**		**Degrees of freedom**
Dataset	**Repeat 1**	**Repeat 2**	**Repeat 3**	
1:3 model	8.807×10^{-3}	5.226×10^{-4}	4.825×10^{-3}	7
1:4 model	8.302×10^{-3}	0.353	7.00×10^{-2}	9
Difference	5.050×10^{-4}	0.352	6.52×10^{-2}	2
Relative difference	0.0608	0.999	0.931	0.2222
Ratio (F)	0.2737	4.493	4.190	
Numerator	2	2	2	
Denominator	9	9	9	
P-value	0.76667	0.04434	0.051753	

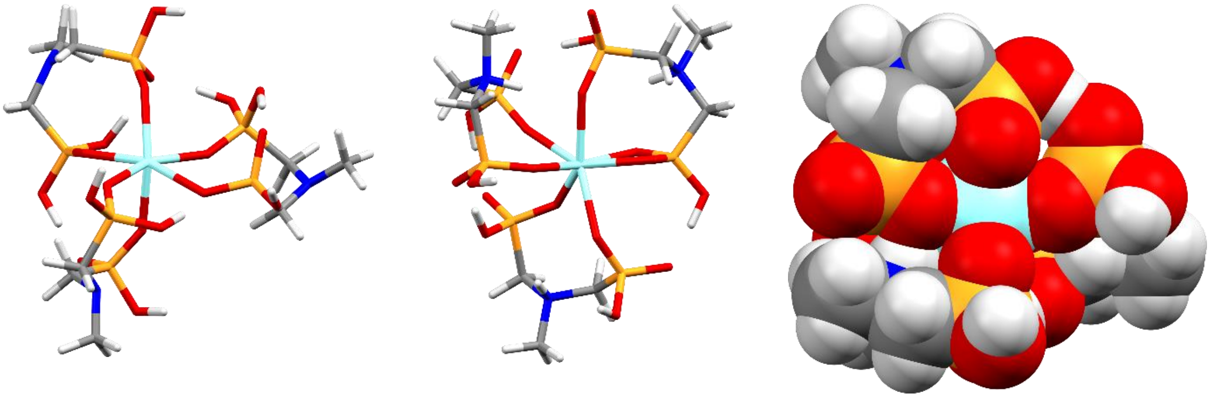

Figure S25. Solution state optimized structures at the PBE1PBE-D3/TZVP level of theory for Y**L**$_3$ in the neutral (left) and zwitterionic (middle) forms as well as the spacefilling model of the zwitterionic form (right). Van der Waals radius of each atom was utilized in the spacefill model.

Table S7. Precipitation percentages for precipitation tests with ammonia. Errors are derived from standard deviation of three replicated measurements.

pH	Sc	La	Ce	Pr	Nd	Sm	Eu	Gd	Tb	Dy	Ho	Er	Y	Tm	Yb	Lu	Th	U
**1**	-11.65 ±6.61	-4.22 ±2.08	-2.72 ±3.41	-3.78 ±3.32	-2.41 ±2.30	-4.42 ±3.12	-2.54 ±2.08	-3.94 ±3.37	-3.98 ±3.05	-3.73 ±3.41	-3.52 ±3.50	-3.98 ±3.71	-3.94 ±2.92	-3.83 ±3.39	-4.46 ±2.22	-4.23 ±3.72	-8.42 ±11.36	-4.64 ±3.78
**1.5**	-6.03 ±3.04	-2.24 ±4.09	-1.32 ±5.26	-1.30 ±3.14	-1.51 ±5.00	-1.30 ±3.27	-1.00 ±2.48	-1.14 ±3.65	-1.46 ±3.29	-1.15 ±3.48	-0.40 ±3.28	-1.03 ±3.49	-1.33 ±3.72	-0.86 ±3.42	-2.43 ±4.16	-1.22 ±3.29	5.28 ±4.24	-0.22 ±2.84
**2**	-9.11 ±7.50	-1.53 ±1.95	-0.65 ±2.82	-1.29 ±3.51	-2.14 ±6.60	-2.25 ±3.90	-0.52 ±1.89	-1.82 ±4.43	-2.00 ±4.65	-1.85 ±4.18	-1.08 ±4.07	-1.71 ±4.21	-1.21 ±5.61	-1.68 ±4.21	-1.73 ±2.11	-2.03 ±4.43	-0.87 ±11.07	-2.59 ±2.81
**2.5**	-7.94 ±6.04	-2.58 ±2.26	-2.23 ±3.34	-0.75 ±2.49	0.24 ±2.48	-1.60 ±2.70	-0.64 ±2.00	-1.05 ±2.71	-1.42 ±2.16	-1.12 ±2.72	-0.23 ±2.73	-0.87 ±2.71	-1.56 ±1.68	-1.06 ±2.78	-3.06 ±2.38	-1.22 ±2.60	11.70 ±11.11	-0.48 ±2.32
**3**	-7.37 ±7.64	-0.88 ±2.48	-2.40 ±3.29	0.40 ±2.90	1.22 ±1.71	-3.56 ±2.66	-0.91 ±2.41	-2.98 ±2.70	-3.34 ±2.34	-3.66 ±3.04	-3.13 ±3.34	-3.45 ±3.23	-0.05 ±4.02	-3.77 ±2.97	-2.09 ±2.45	-2.83 ±2.73	37.32 ±15.24	-0.93 ±2.73
**3.5**	-2.18 ±9.47	0.01 ±2.13	-3.33 ±4.13	2.12 ±3.63	4.11 ±4.02	-2.71 ±3.67	-0.82 ±1.33	-3.11 ±3.83	-3.37 ±3.58	-4.16 ±3.56	-3.55 ±4.00	-3.39 ±3.53	2.30 ±2.99	-4.43 ±3.80	-1.99 ±2.87	-2.53 ±3.42	78.08 ±4.45	3.90 ±2.13
**4**	8.86 ±8.94	0.02 ±1.53	-4.13 ±2.80	1.81 ±1.99	-0.40 ±8.96	-3.59 ±2.47	-0.61 ±2.60	-4.06 ±2.85	-4.30 ± 2.32	-5.11 ±2.70	-4.65 ±3.23	-4.37 ±2.55	2.59 ±1.82	-5.23 ±2.88	-2.37 ±1.75	-3.60 ±2.50	91.20 ±0.78	13.13 ±3.33

Table S8. Precipitation percentages for precipitation tests performed with ligand **1**. Errors are derived from standard deviations of three replicated measurements.

pH	Sc	La	Ce	Pr	Nd	Sm	Eu	Gd	Tb	Dy	Ho	Er	Y	Tm	Yb	Lu	Th	U
**1**	0.74 ±2.61 %	-10.25 ±3.50 %	-8.53 ±4.41 %	-13.83 ±6.91 %	-11.24 ±0.44 %	-9.58 ±2.19 %	-5.37 ±5.56 %	-10.33 ±7.94 %	-13.94 ±1.48 %	-25.30 ±10.82 %	-5.25 ±4.46 %	-9.29 ±4.69 %	-7.53 ±6.40 %	-7.60 ±7.01 %	-7.30 ±7.43 %	-10.20 ±5.33 %	-0.03 ±11.97 %	10.25 ±0.18 %
**1.5**	6.60 ±9.45 %	-6.49 ±1.42 %	-1.98 ±2.35 %	-6.49 ±5.56 %	-4.81 ±2.93 %	-3.84 ±1.00 %	1.74 ±3.05 %	-4.03 ±5.08 %	-8.00 ±3.52 %	-18.53 ±9.94 %	1.16 ±1.73 %	-2.76 ±1.74 %	-2.58 ±3.83 %	-1.11 ±4.08 %	-0.99 ±4.75 %	-2.98 ±2.96 %	6.52 ±8.10 %	1.33 ±0.23 %
**2**	10.65 ±8.74 %	-2.64 ±4.77 %	0.37 ±5.10 %	-5.06 ±6.77 %	1.44 ±2.06 %	-0.82 ±3.20 %	1.81 ±6.43 %	-1.58 ±8.05 %	-4.89 ±1.67 %	-15.62 ±9.55 %	3.22 ±5.74 %	-0.14 ±5.44 %	0.39 ±6.09 %	1.24 ±7.58 %	2.88 ±8.42 %	0.24 ±6.76 %	9.01 ±8.54 %	1.48 ±2.37 %
**2.5**	36.35 ±3.40 %	-0.07 ±3.08 %	1.47 ±3.42 %	-2.43 ±6.58 %	-0.46 ±0.51 %	0.68 ±1.35 %	3.75 ±4.37 %	0.41 ±6.32 %	-3.01 ±1.43 %	-12.88 ±9.56 %	6.00 ±3.83 %	2.69 ±3.02 %	1.71 ±4.48 %	5.64 ±5.06 %	10.58 ±4.27 %	10.95 ±1.56 %	35.74 ±4.62 %	44.25 ±1.65 %
**3**	60.45 ±15.35 %	0.24 ±3.44 %	3.25 ±3.36 %	0.71 ±6.27 %	2.84 ±0.45 %	0.30 ±1.65 %	6.13 ±3.79 %	0.52 ±6.51 %	-1.63 ±1.71 %	-11.29 ±9.81 %	8.02 ±2.30 %	8.12 ±1.66 %	5.26 ±3.04 %	18.61 ±1.87 %	36.97 ±7.94 %	43.28 ±13.70 %	56.98 ±15.38 %	91.09 ±0.89 %
**3.5**	84.37 ±6.01 %	14.49 ±8.34 %	20.97 ±8.72 %	20.09 ±10.9 %	21.25 ±5.35 %	20.78 ±9.48 %	26.37 ±10.35 %	20.24 ±12.55 %	21.89 ±8.43 %	15.72 ±3.38 %	31.66 ±9.68 %	35.10 ±8.41 %	30.06 ±10.11 %	49.39 ±5.87 %	65.76 ±0.67 %	71.91 ±2.92 %	78.85 ±6.11 %	101.21 ±0.14 %
**4**	82.16 ±7.96 %	15.92 ±6.65 %	21.28 ±5.46 %	19.42 ±6.50 %	19.55 ±1.40 %	19.47 ±4.13 %	24.17 ±6.32 %	19.71 ±7.61 %	19.74 ±2.44 %	13.71 ±8.25 %	30.48 ±4.97 %	33.97 ±3.33 %	29.18 ±5.64 %	47.04 ±0.86 %	61.75 ±3.48 %	67.66 ±7.15 %	76.65 ±8.22 %	100.52 ±0.60 %

Table S9. Precipitation percentages for precipitation tests performed with ligand **2**. Errors are derived from standard deviations of three replicated measurements.

pH	Sc	La	Ce	Pr	Nd	Sm	Eu	Gd	Tb	Dy	Ho	Er	Y	Tm	Yb	Lu	Th	U
**1**	22.89 ±3.62 %	5.88 ±3.21%	7.40 ±3.72%	2.37 ±4.43%	7.64 ±2.37 %	6.13 ±1.46 %	4.82 ±3.64%	2.53 ±4.16%	3.76 ±1.80%	-3.24 ±8.78%	6.96 ±1.26%	5.00 ±2.25 %	3.59 ±1.68 %	4.53 ±2.82%	5.82 ±2.46%	4.40 ±2.70 %	27.24 ±1.00 %	10.50 ±0.99%
**1.5**	42.57 ±17.35 %	4.70 ±4.42%	4.77 ±2.79%	1.07 ±6.37%	5.18 ±2.28 %	5.25 ±2.82 %	4.94 ±2.62%	2.76 ±6.45%	3.97 ±4.33%	-3.69 ±11.58%	6.32 ±1.89%	4.89 ±4.19 %	4.74 ±2.58 %	5.14 ±5.14%	4.77 ±4.49%	4.92 ±4.90 %	45.72 ±12.54 %	15.60 ±4.46%
**2**	45.45 ±8.64%	6.42 ±1.73%	9.04 ±1.43%	4.71 ±1.57%	5.44 ±1.56 %	4.70 ±0.88 %	8.52 ±2.20%	2.92 ±2.53%	3.77 ±0.38%	-3.73 ±7.53%	6.53 ±1.77%	5.15 ±1.26 %	5.76 ±0.90 %	5.27 ±1.34%	9.19 ±1.66%	6.53 ±1.13 %	46.76 ±5.74 %	24.82 ±1.22%
**2.5**	53.60 ±7.82%	12.53 ±1.80%	12.03 ±1.37 %	9.77 ±1.80%	12.27 ±1.09 %	11.06 ±2.69 %	14.42 ±2.62%	8.97 ±3.74%	10.79 ±2.80%	3.25 7.49%	12.86 ±3.32%	12.08 ±3.30 %	12.03 ±1.87 %	12.50 ±3.19%	17.76 ±2.68%	16.73 ±3.31 %	53.80 ±5.25 %	37.44 ±2.04%
**3**	58.83 ±9.82%	11.52 ±1.06%	12.90 ±1.50%	10.51 ±2.68%	10.79 ±1.02 %	11.15 ±1.35 %	14.19 ±1.89%	8.62 ±3.32%	10.46 1.87%	3.32 8.16%	13.75 ±2.20%	13.10 ±2.48 %	12.19 ±1.15 %	15.91 ±3.36%	25.14 ±3.75%	26.76 ±5.04 %	58.36 ±6.76 %	43.90 ±0.54%
**3.5**	64.49 ±6.44%	9.21 ±0.68%	12.82 ±1.83%	8.90 ±3.92%	9.72 ±2.04 %	10.34 ±1.59 %	14.11 0.74%	8.02 ±3.95%	10.69 2.27%	3.71 9.62%	14.64 ±1.05%	16.01 ±2.75 %	13.56 ±1.62 %	22.42 ±3.03%	35.17 ±1.59%	38.49 ±2.52 %	62.11 ±4.28 %	60.62 ±0.91%
**4**	70.46 ±4.00%	10.09 ±2.64%	13.96 ±1.57%	11.61 3.76%	14.67 ±2.12 %	12.71 1.89%	15.95 1.15%	10.98 2.08%	13.42 0.82%	7.86 7.75%	18.88 ±1.21%	20.97 0.93%	17.12 ±1.37 %	29.10 ±1.46%	41.81 ±0.42%	46.50 ±0.52 %	67.86 ±1.99 %	72.37 ±1.53%

Table S10. Precipitation percentages for precipitation tests performed with ligand **3**. Errors are derived from standard deviations of three replicated measurements.

pH	Sc	La	Ce	Pr	Nd	Sm	Eu	Gd	Tb	Dy	Ho	Er	Y	Tm	Yb	Lu	Th	U
**1**	35.75 ±7.41%	-7.56 ±6.61%	-5.87 4.58%	-11.27 ±6.78%	-5.17 ±4.68%	-9.07 ±7.81%	-6.25 ±6.85%	-9.91 ±5.09%	-9.00 ±5.75%	-14.05 ±6.06 %	-6.78 ±6.91%	-7.84 ±5.51%	-8.65 ±5.79%	-8.85 ±6.03%	-8.84 ±5.95%	-8.59 ±6.13%	36.39 ±5.65%	10.47 ±0.33%
**1.5**	47.10 ±12.34%	-2.12 ±0.53%	0.43 ±2.37%	-4.75 ±2.70%	-2.55 ±1.38%	-3.40 ±1.68%	-0.99 ±1.09%	-5.33 ±1.96%	-4.09 ±0.31%	-9.32 ±5.91%	-1.68 ±1.62%	-2.75 ±0.16%	-4.55 ±0.94%	-3.34 ±0.52%	-0.70 ±0.88%	-2.49 ±0.14%	47.68 ±11.05%	15.85 ±0.46%
**2**	59.89 ±7.79%	2.49 ±1.00%	7.09 ±3.36%	0.04 ±1.87%	2.55 ±1.12%	0.01 ±2.50%	4.11 ±2.03%	-2.02 ±3.98%	-0.68 ±3.24%	-6.51 ±7.31%	1.30 ±2.90%	0.33 ±3.29%	-3.01 ±3.33%	0.20 ±3.23%	5.64 ±1.84%	3.29 ±2.83%	56.65 ±7.01%	30.77 ±0.01%
**2.5**	68.79 ±4.30%	5.43 ±3.35%	5.11 ±1.47%	3.14 ±2.66%	6.11 ±2.73%	3.18 ±4.13%	7.30 ±4.21%	2.01 ±5.85%	3.24 ±5.54%	-2.24 ±8.94%	5.48 ±4.88%	5.18 ±5.23%	-2.80 ±7.53%	6.27 ±5.11%	13.23 ±3.64%	13.37 ±3.62%	64.87 ±3.83%	53.16 ±0.57%
**3**	79.11 ±0.93%	3.94 ±1.66%	6.04 ±0.45%	4.67 ±2.20%	4.85 ±0.58%	4.53 ±1.10%	7.83 ±2.11%	3.26 ±3.31%	5.39 ±1.39%	0.59 ±7.29%	9.04 ±0.93%	10.30 ±1.09%	-32.56 ±19.88%	16.15 ±1.15%	30.02 ±3.71%	36.46 ±5.08%	74.53 ±1.70%	82.40 ±0.81%
**3.5**	80.95 ±0.76%	6.15 ±4.30%	11.51 ±4.19%	10.31 ±5.08%	12.39 ±4.67%	11.36 ±5.05%	13.92 ±4.86%	9.01 ±3.89%	12.57 ±3.96%	9.03 ±5.96%	17.27 ±5.67%	20.26 ±4.91%	6.22 ±1.44%	29.66 ±5.85%	43.67 ±6.68%	49.41 ±6.91%	76.57 ±0.87%	86.55 ±0.70%
**4**	81.01 ±0.07%	11.74 ±5.75%	15.09 ±8.38%	15.41 ±5.44%	-57.73 ±30.61%	17.63 ±5.58%	18.94 ±5.25%	14.08 ±3.34%	17.41 ±3.69%	14.36 ±4.59%	23.59 ±5.30%	26.39 ±4.62%	12.85 ±1.44%	36.01 ±5.31%	48.34 ±6.47%	53.15 ±6.63%	78.23 ±0.49%	75.14 ±1.58%

Table S11. Precipitation percentages for precipitation tests performed with ligand **4**. Errors are derived from standard deviations of three replicated measurements.

pH	Sc	La	Ce	Pr	Nd	Sm	Eu	Gd	Tb	Dy	Ho	Er	Y	Tm	Yb	Lu	Th	U
**1**	94.39 ±1.47%	3.85 ±3.80%	5.85 ±2.32%	2.42 ±1.02%	3.03 ±2.74%	-0.02 ±3.01%	1.73 ±3.46%	0.26 ±4.15%	1.15 ±3.75%	-2.09 ±5.07%	1.83 ±3.73%	1.79 ±3.82%	0.83 ±4.62%	1.03 3.69%	1.74 ±3.37%	3.15 ±3.78%	87.67 ±0.27%	29.77 ±2.33%
**1.5**	98.42 ±0.56%	12.65 ±2.94%	14.80 ±4.28%	14.62 ±4.81%	17.76 ±2.48%	15.88 ±4.53%	16.27 ±4.81%	15.41 ±2.94%	17.44 ±4.17%	15.76 ±3.46%	19.53 ±5.08%	20.00 ±4.97%	16.46 ±3.62%	20.80 ±5.20%	21.60 ±6.85%	24.50 ±6.08%	91.59 ±1.31%	73.98 ±1.25%
**2**	99.51 ±0.88%	14.93 ±2.62%	18.79 ±1.51%	16.38 ±3.07%	18.74 ±1.53%	18.62 ±2.28%	19.60 ±3.28%	18.68 ±1.14%	20.81 ±2.62%	19.31 ±2.35%	22.99 ±3.61%	23.72 ±3.29%	19.77 ±2.20%	25.06 ±3.44%	26.45 ±4.50%	29.73 ±3.70%	92.67 ±0.87%	80.53 ±0.46%
**2.5**	100.06 ±0.72%	5.74 ±3.11%	9.35 ±3.34%	4.90 ±0.75%	6.65 ±2.29%	5.09 ±2.49%	6.32 ±3.11%	4.79 ±3.20%	6.33 ±2.78%	3.22 ±4.73%	8.04 ±3.08%	8.87 ±2.84%	5.20 ±3.84%	11.29 ±2.57%	17.23 ±2.71%	22.70 ±2.41%	91.58 ±0.69%	69.60 ±1.16%
**3**	100.12 ±0.62%	9.01 ±3.22%	14.03 ±1.74%	13.19 ±0.81%	15.45 ±1.93%	16.58 ±2.17%	18.60 ±3.32%	16.22 ±3.50%	20.80 ±2.82%	20.13 ±4.95%	25.33 ±2.35%	29.92 ±2.18%	19.61 ±3.47%	42.87 ±1.07%	62.74 ±0.90%	72.31 ±1.56%	93.19 ±0.65%	102.60 ±2.38%
**3.5**	100.29 ±0.62%	20.15 ±3.05%	29.47 ±1.79%	32.80 ±2.10%	32.96 ±2.82%	36.63 ±3.49%	39.25 ±4.02%	34.32 ±4.70%	42.05 ±4.23%	43.39 ±5.65%	48.88 ±3.83%	56.05 ±3.47%	42.89 ±4.42%	71.80 ±1.56%	87.56 ±0.60%	92.65 ±0.75%	94.90 ±0.41%	108.49 ±1.73%
**4**	99.74 ±0.53%	38.12 ±4.24%	48.14 ±1.93%	52.59 ±3.52%	52.85 ±3.53%	56.03 ±3.34%	56.21 ±4.44%	52.33 ±4.34%	59.53 ±3.95%	61.08 ±4.80%	66.32 ±3.10%	72.81 ±2.86%	60.45 ±4.78%	84.04 ±0.61%	93.56 ±0.26%	96.16 ±0.79%	95.71 ±0.64%	107.32 ±2.32%

Table S12. Precipitation percentages for precipitation tests performed with ligand **5**. Errors are derived from standard deviations of three replicated measurements.

pH	Sc	La	Ce	Pr	Nd	Sm	Eu	Gd	Tb	Dy	Ho	Er	Y	Tm	Yb	Lu	Th	U
**1**	100.27 ±0.35%	9.81 ±3.54%	10.92 ±3.39%	10.80 ±3.94%	11.62 ±3.25%	7.23 ±3.46%	8.66 ±3.10%	8.81 ±2.69%	8.77 ±3.03%	8.64 ±2.83%	8.86 ±2.57%	9.25 ±2.58%	8.14 ±3.10%	9.52 ±2.55%	8.44 ±3.05%	10.20 ±2.80%	92.42 ±0.56%	47.71 ±2.46%
**1.5**	100.42 ±0.31%	8.61 ±2.63%	9.56 ±1.83%	9.07 ±2.97%	10.25 ±2.07%	5.79 ±2.87%	7.56 ±2.49%	7.69 ±2.32%	7.30 ±2.57%	7.31 ±2.39%	7.83 ±2.11%	7.95 ±2.15%	7.02 ±2.41%	8.43 ±2.21%	8.07 ±2.26%	10.36 ±2.16%	92.07 ±0.38%	53.83 ±2.18%
**2**	100.85 ±0.33%	10.36 ±2.58%	13.28 ±1.84%	11.39 ±2.82%	11.98 ±2.12%	8.74 ±2.25%	9.16 ±2.52%	10.51 ±2.01%	10.21 ±2.14%	10.89 ±2.05 %	11.09 ±1.99%	12.07 ±1.88%	10.03 ±2.34%	14.30 ±1.81%	16.65 ±2.14%	22.68 ±1.79%	92.89 ±0.71%	69.56 ±1.43%
**2.5**	101.00 ±0.34%	14.19 ±2.57%	17.55 ±1.81%	18.88 ±2.00%	18.68 ±1.65%	13.56 ±2.30%	17.43 ±2.22%	14.82 ±2.55%	16.39 ±2.79%	18.09 ±2.67%	18.60 ±3.17%	21.40 ±3.30%	19.88 ±2.41%	28.97 ±3.85%	42.69 ±3.95%	49.11 ±4.43%	93.52 ±0.84%	95.04 ±0.23%
**3**	100.04 ±0.39%	21.29 ±2.92%	30.81 ±2.44%	35.80 ±2.20%	32.79 ±2.21%	37.51 ±3.27%	38.37 ±4.15%	36.75 ±3.44%	43.29 ±3.76%	47.48 ±3.57%	48.88 ±3.98%	56.43 ±3.65%	43.52 ±4.26%	72.47 ±2.01%	87.74 ±1.22%	92.98 ±0.22%	94.60 ±0.22%	106.33 ±5.17%
**3.5**	100.27 ±0.29%	53.20 ±2.09%	63.95 ±2.49%	69.77 ±1.99%	67.46 ±1.09%	71.70 ±0.78%	71.41 ±1.59%	69.08 ±1.03%	75.60 ±0.99%	78.73 ±0.56%	80.35 ±0.45%	85.74 ±0.45%	76.53 ±1.14%	93.73 ±1.33%	98.06 ±0.41%	98.81 ±0.80%	97.32 ±0.68%	102.69 ±2.20%
**4**	100.02 ±0.41%	69.30 ±1.99%	79.16 ±0.50%	82.62 ±1.08%	80.34 ±1.36%	82.54 ±1.04%	82.05 ±2.72%	78.80 ±2.74%	83.97 ±2.38%	86.59 ±1.57%	88.22 ±1.44%	92.29 ±0.75%	85.97 ±2.24%	97.03 ±0.81%	99.29 ±0.20%	99.43 ±0.57%	98.28 ±0.07%	102.06 ±1.68%

Table S13. Precipitation percentages for precipitation tests performed with ligand **6**. Errors are derived from standard deviations of three replicated measurements.

pH	Sc	La	Ce	Pr	Nd	Sm	Eu	Gd	Tb	Dy	Ho	Er	Y	Tm	Yb	Lu	Th	U
**1**	100.08 ±0.39%	6.80 ±5.40%	9.06 ±5.39%	8.87 ±3.57%	8.97 4.97%	5.17 ±4.97%	9.13 ±4.97%	7.45 ±5.57%	8.95 ±5.16%	9.02 ±4.91%	8.39 ±4.85%	9.33 ±5.14%	7.51 ±4.66%	10.36 ±4.48%	12.75 ±3.13%	12.96 ±3.53%	92.69 ±0.68%	108.42 ±0.40%
**1.5**	100.85 ±0.09%	10.37 ±3.51%	13.98 ±1.52%	17.77 ±1.55%	17.16 ±1.69%	17.74 ±1.69%	22.34 ±2.38%	20.30 ±1.86%	24.00 ±3.09%	25.23 ±3.76%	24.35 ±3.62%	26.65 ±3.83%	21.70 ±2.51%	31.04 ±6.17%	37.95 ±8.87%	40.33 ±9.25%	93.49 ±0.84%	114.87 ±0.70%
**2**	100.73 ±0.22%	7.41 ±3.32%	12.33 ±1.86%	15.44 ±2.74%	15.18 ±1.49%	17.40 ±2.71%	21.65 ±3.52%	20.28 ±3.02%	24.72 ±4.57%	26.33 ±5.59%	25.69 ±5.70%	29.07 ±5.56%	21.47 ±3.81%	36.12 ±8.21%	46.60 ±9.53%	51.86 ±9.30%	93.46 ±1.01%	115.30 ±0.72%
**2.5**	100.67 ±0.33%	15.68 ±2.47%	23.43 ±5.32%	29.67 ±9.92%	28.18 ±7.85%	37.05 ±11.57%	40.53 ±12.29%	39.12 ±11.83%	44.78 ±13.87%	46.62 ±14.86%	46.08 ±14.88%	49.92 ±14.75%	40.69 ±13.13%	57.83 ±17.00%	66.31 ±16.51%	70.91 ±15.82%	94.77 ±1.66%	113.25 ±0.62%
**3**	100.46 ±0.24%	35.00 ±8.70%	47.65 ±11.52%	56.04 ±13.02%	53.12 ±13.63%	63.45 ±15.06%	65.19 ±14.38%	63.35 ±15.18%	69.31 ±14.45%	71.48 ±14.19%	71.58 ±13.76%	75.83 ±12.02%	66.65 ±13.29%	83.70 ±9.35%	90.44 ±5.28%	92.96 ±4.05%	96.74 ±2.07%	109.04 ±0.43%
**3.5**	100.37 ±0.27%	54.09 ±2.58%	68.61 ±0.69%	75.80 ±1.74%	73.96 ±1.75%	82.39 ±3.23%	83.09 ±4.34%	81.46 ±4.70%	86.00 ±4.26%	87.41 ±4.15%	87.65 ±3.68%	90.13 ±3.18%	84.44 ±2.88%	94.65 ±2.34%	96.89 ±1.44%	97.69 ±1.17%	98.53 ±0.91%	106.51 ±0.31%
**4**	99.97 ±0.53%	71.70 ±4.2%	82.69 ±0.65%	87.75 ±0.25%	86.94 ±0.25%	90.88 ±1.75%	90.95 ±2.33%	89.96 ±2.44%	92.48 ±2.39%	93.30 ±2.29%	93.92 ±1.92%	94.82 ±1.71%	91.52 ±0.68%	97.22 ±1.46%	97.90 ±1.29%	98.57 ±1.18%	99.05 ±0.95%	105.42 ±0.26%

Table S14. Separation factors between REEs. thorium and uranium for ligand **1.** Hyphen (-) and star (*) means that no separation factor could be calculated because of complete metal precipitation (*) no precipitation at all (-).

pH	Sc/La	Ce/La	Pr/Ce	Pr/Nd	Nd/Sm	Sm/Eu	Eu/Gd	Gd/Tb	Tb/Dy	Dy/Ho	Ho/Er	Er/Y	Y/Tm	Tm/Yb	Yb/Lu	Lu/Th	Th/U	U/Lu
**1**	-	-	-	-	-	-	-	-	-	-	-	-	-	-	-	-	-	-
**1.5**	-	-	-	-	-	-	-	-	-	-	-	-	-	-	-	-	-	-
**2**	-	-	-	-	-	-	-	-	-	-	-	-	-	-	-	-	-	-
**2.5**	-	-	-	-	-	-	-	-	-	-	41.36 ±57.04	-	-	1.78 ±0.93	2.52 ±0.91	4.50 ±0.33	1.28 ±0.01	5.76 ±0.44
**3**	-	-	2.06 ±0.58	1.16 ±0.10	-	-	-	-	2.67 ±0.83	5.25 ±5.71	2.00 ±0.20	3.02 ±0.42	8.88 ±1.23	2.88 ±0.15	1.74 ±0.03	1.80 ±0.12	4.14 ±0.60	7.49 ±1.56
**3.5**	154.2 ±113.6	2.56 ±1.59	1.17 ±0.12	1.12 ±0.04	1.41 ±0.02	1.28 ±0.16	1.22 ±0.04	1.00 ±0.38	1.13 ±0.21	1.28 ±0.19	1.47 ±0.10	1.50 ±0.08	3.17 ±0.32	2.25 ±0.10	1.68 ±0.09	1.76 ±0.09	*	*
**4**	101.7 ±80.9	2.12 ±1.12	1.08 ±0.19	1.07 ±0.01	1.25 ±0.04	1.08 ±0.20	1.04 ±0.16	1.01 ±0.26	1.14 ±0.19	1.25 ±0.16	1.43 ±0.12	1.72 ±0.14	3.34 ±0.16	2.03 ±0.07	1.67 ±0.03	1.75 ±0.04	*	*

Table S15. Separation factors between REEs. thorium and uranium for ligand **2.** Hyphen (-) and star (*) means that no separation factor could be calculated because of complete metal precipitation (*) no precipitation at all (-).

pH	Sc/La	Ce/La	Pr/Ce	Pr/Nd	Nd/Sm	Sm/Eu	Eu/Gd	Gd/Tb	Tb/Dy	Dy/Ho	Ho/Er	Er/Y	Y/Tm	Tm/Yb	Yb/Lu	Lu/Th	Th/U	U/Lu
**1**	7.94 ±6.21	2.53 ±2.68	1.23 ±0.09	0.92 ±0.03	1.04 ±0.04	11.28 ±11.26	8.80 ±8.76	1.05 ±0.06	1.16 ±0.01	1.24 ±0.29	1.09 ±0.19	2.75 ±2.63	2.76 ±2.46	10.34 ±12.79	9.07 ±10.85	4.62 ±3.56	2.01 ±0.83	2.88 ±0.19
**1.5**	-	-	-	0.49 ±0.69	1.19 ±0.29	1.00 ±1.41	-	-		-	-	1.11 ±1.56	3.10 ±1.27	1.03 ±1.45	-	121.95 ±166.91	1.29 ±0.44	76.72 ±103.03
**2**	6.82 ±3.75	1.18 ±0.05	1.14 ±0.13	1.48 ±0.51	1.40 ±0.57	1.45 ±0.12	1.52 ±0.01	1.00 ±0.04	1.21 ±0.21	1.36 ±0.39	0.98 ±0.19	0.71 ±0.10	0.88 ±0.03	1.40 ±0.10	0.85 ±0.01	6.02 ±0.09	1.53 ±0.92	4.03 ±1.84
**2.5**	6.28 ±2.58	1.15 ±0.04	1.07 ±0.05	1.09 ±0.07	1.50 ±0.64	1.37 ±0.42	1.43 ±0.47	1.03 ±0.11	1.20 ±0.16	1.17 ±0.23	1.04 ±0.02	0.90 ±0.29	1.03 ±0.26	1.44 ±0.43	1.01 ±0.18	3.86 ±0.09	1.40 ±0.50	3.14 ±1.38
**3**	7.23 ±2.54	1.13 ±0.10	1.15 ±0.14	1.18 ±0.13	1.18 ±0.16	1.24 ±0.07	1.31 ±0.06	0.99 ±0.08	1.16 ±0.12	1.25 ±0.28	1.03 ±0.06	1.00 ±0.11	1.28 ±0.05	1.55 ±0.10	1.14 ±0.02	2.61 ±0.47	1.32 ±0.09	2.49 ±0.52
**3.5**	12.64 ±0.85	1.33 ±0.14	1.14 ±0.11	1.22 ±0.27	1.21 ±0.29	1.40 ±0.13	1.38 ±0.08	0.97 ±0.17	1.23 ±0.13	1.41 ±0.49	1.06 ±0.23	1.15 ±0.08	1.85 ±0.17	1.77 ±0.03	1.22 ±0.02	1.77 ±0.33	1.21 ±0.50	2.37 ±0.03
**4**	12.48 ±0.63	1.28 ±0.05	1.06 ±0.08	1.02 ±0.01	1.14 ±0.10	1.09 ±0.03	1.14 ±0.18	0.98 ±0.12	1.15 ±0.01	1.27 ±0.31	1.06 ±0.24	1.25 ±0.22	1.94 ±0.33	1.65 ±0.12	1.30 ±0.09	1.70 ±0.22	1.42 ±0.93	2.81 ±0.54

Table S16. Separation factors between REEs. thorium and uranium for ligand **3.** Hyphen (-) and star (*) means that no separation factor could be calculated because of complete metal precipitation (*) no precipitation at all (-).

pH	Sc/La	Ce/La	Pr/Ce	Pr/Nd	Nd/Sm	Sm/Eu	Eu/Gd	Gd/Tb	Tb/Dy	Dy/Ho	Ho/Er	Er/Y	Y/Tm	Tm/Yb	Yb/Lu	Lu/Th	Th/U	U/Lu
**1**	-	-	-	-	-	-	-	-	-	-	-	-	-	-	-	-	2.40 ±1.78	-
**1.5**	-	-	-	-	-	-	-	-	-	-	-	-	-	-	-	-	2.15 ±1.05	-
**2**	107.2 ±138.3	2.92 ±2.66	1.28± 0.37	1.75 ±0.98	-	-	-	-	-	-	-	-	-	-	0.75 ±1.07	-	1.76 ±0.78	-
**2.5**	339.3 ±458.8	5.19 ±5.76	1.36± 0.50	1.29 ±0.21	-	-	-	-	-	-	-	-	-	12.32 ±14.70	1.07 ±0.13	9.17 ±1.85	1.35 ±0.26	8.68 ±5.04
**3**	343.7 ±448.6	4.88 ±5.31	1.10± 0.25	1.39 ±0.36	1.44 ±0.31	1.76 ±1.02	1.41 ±0.78	0.84 ±0.12	1.51 ±0.05	1.44 ±0.58	1.18 ±0.45	-	-	2.22 ±0.23	1.18 ±0.57	3.44 ±0.25	1.42 ±0.97	5.92 ±1.34
**3.5**	30.88 ±17.24	1.34 ±0.64	1.08± 0.22	1.08 ±0.08	1.06 ±0.12	1.18 ±0.25	1.23 ±0.21	0.86 ±0.04	1.16 ±0.08	1.26 ±0.24	1.07 ±0.28	3.33 ±1.17	5.44 ±1.95	1.73 ±0.07	1.08 ±0.38	2.05 ±0.13	1.60 ±1.48	4.54 ±0.92
**4**	17.62 ±8.04	1.24 ±0.53	1.07± 0.004	-	-	1.05 ±0.11	1.22 ±0.04	0.90 ±0.04	1.18 ±0.13	1.27 ±0.21	1.07 ±0.25	2.46 ±0.48	3.90 ±0.94	1.64 ±0.04	1.03 ±0.31	1.94 ±0.05	1.26 ±0.07	1.91 ±0.56

Table S17. Separation factors between REEs. thorium and uranium for ligand **4.** Hyphen (-) and star (*) means that no separation factor could be calculated because of complete metal precipitation (*) no precipitation at all (-).

pH	Sc/La	Ce/La	Pr/Ce	Pr/Nd	Nd/Sm	Sm/Eu	Eu/Gd	d/Tb	Tb/Dy	Dy/Ho	Ho/Er	Er/Y	Y/Tm	Tm/Yb	Yb/Lu	Lu/Th	Th/U	U/Lu
**1**	-	-	-	-	-	-	-	-	-	-	-	-	-	-	-	-	19.97 ±12.93	-
**1.5**	756.6 ±615.6	1.63 ±0.39	1.15 ±0.10	1.02 ±0.01	1.34 ±0.23	1.06 ±0.08	0.98 ±0.01	1.18 ±0.04	1.25 ±0.19	1.15 ±0.18	1.22 ±0.14	1.45 ±0.03	1.48 ±0.24	1.12 ±0.15	1.19 ±0.02	27.23 ±3.85	4.30 ±1.63	6.64 ±1.62
**2**	1322.8 ±1289.9	1.62 ±0.33	1.09 ±0.20	1.10 ±0.01	1.23 ±0.29	0.99 ±0.04	0.98 ±0.08	1.19 ±0.07	1.31 ±0.21	1.20 ±0.23	1.20 ±0.14	1.46 ±0.04	1.53 ±0.29	1.11 ±0.20	1.21 ±0.09	26.31 ±4.58	3.20 ±0.88	8.34 ±0.87
**2.5**	*	-	-	-	-	4.92 ±4.87	-	-	-	-	-	-	-	3.60 ±2.97	1.65 ±0.14	44.41 ±6.34	4.79 ±1.31	9.45 ±1.26
**3**	*	3.81 ±0.86	1.23 ±0.31	1.10 ±0.08	1.70 ±0.95	1.00 ±0.08	1.11 ±0.27	1.49 ±0.17	1.50 ±0.29	1.47 ±0.73	1.53 ±0.22	2.21 ±0.15	4.15 ±0.75	2.46 ±0.42	1.70 ±0.08	4.70 ±1.02	*	*
**3.5**	*	1.98 ±0.28	1.26 ±0.15	1.09 ±0.11	1.21 ±0.40	0.95 ±0.01	1.21 ±0.06	1.43 ±0.06	1.19 ±0.05	1.19 ±0.35	1.46 ±0.02	1.84 ±0.01	3.91 ±0.38	2.84 ±0.22	2.16 ±0.02	1.22 ±0.08	*	*
**4**	*	1.72 ±0.14	1.17 ±0.12	1.11 ±0.02	1.07 ±0.27	1.07 ±0.01	1.10 ±0.02	1.34 ±0.04	1.15 ±0.09	1.15 ±0.35	1.42 ±0.01	1.90 ±0.02	4.10 ±0.37	2.75 ±0.10	2.32 ±0.02	0.73 ±0.20	*	*

Table S18. Separation factors between REEs. thorium and uranium for ligand **5.** Hyphen (-) and star (*) means that no separation factor could be calculated because of complete metal precipitation (*) no precipitation at all (-).

pH	Sc/La	Ce/La	Pr/Ce	Pr/Nd	Nd/Sm	Sm/Eu	Eu/Gd	Gd/Tb	Tb/Dy	Dy/Ho	Ho/Er	Er/Y	Y/Tm	Tm/Yb	Yb/Lu	Lu/Th	Th/U	U/Lu
**1**	15161.2 ±530.8	-	-	-	-	-	-	-	-	-	-	-	-	-	-	-	14.72 ±0.26	7.54 ±0.15
**1.5**	*	-	-	-	-	-	-	-	-	-	-	-	-	-	-	-	10.61 ±0.54	10.78 ±0.14
**2**	*	-	0.56 ±0.79	-	-	-	-	-	-	-	-	-	-	3.24 ±2.97	1.91 ±0.37	48.32 ±6.04	6.09 ±0.36	8.54 ±0.53
**2.5**	*	-	2.18 ±1.49	1.54 ±0.65	2.04 ±0.10	1.01 ±0.54	1.42 ±0.04	1.14 ±0.36	1.06 ±0.33	1.12 ±0.15	1.28 ±0.04	1.30 ±0.37	2.31 ±0.92	2.05 ±0.14	1.36 ±0.06	11.72 ±4.24	0.84 ±0.03	23.79 ±0.24
**3**	1621.3 ±179.4	2.11 ±0.21	1.37 ±0.11	1.29 ±0.18	1.32 ±0.66	1.03 ±0.01	1.08 ±0.07	1.08 ±0.47	1.02 ±0.27	1.04 ±0.11	1.38 ±0.01	1.76 ±0.05	3.63 ±0.38	2.84 ±0.20	1.76 ±0.54	1.12 ±0.15	*	*
**3.5**	5832.0 ±353.6	1.61 ±0.14	1.38 ±0.15	1.14 ±0.05	1.12 ±0.41	1.13 ±0.01	1.13 ±0.08	1.08 ±0.50	0.98 ±0.24	0.99 ±0.13	1.45 ±0.11	1.81 ±0.32	4.61 ±2.45	3.45 ±0.17	2.52 ±2.12	0.39 ±0.36	*	*
**4**	112.0 ±17.5	1.68 ±0.24	1.33 ±0.20	1.19 ±0.02	1.03 ±0.25	1.20 ±0.05	1.25 ±0.07	1.09 ±0.54	0.95 ±0.24	1.02 ±0.22	1.57 ±0.13	1.87 ±0.37	5.57 ±4.73	4.33 ±0.04	*	*	*	*

Table S19. Separation factors between REEs. thorium and uranium for ligand **6.** Hyphen (-) and star (*) means that no separation factor could be calculated because of complete metal precipitation (*) no precipitation at all (-).

pH	Sc/La	Ce/La	Pr/Ce	Pr/Nd	Nd/Sm	Sm/Eu	Eu/Gd	Gd/Tb	Tb/Dy	Dy/Ho	Ho/Er	Er/Y	Y/Tm	Tm/Yb	Yb/Lu	Lu/Th	Th/U	U/Lu
**1**	*	-	-	-	-	-	-	-	-	-	-	-	-	-	-	-		*
**1.5**	*	-	-	-	-	-	-	-	-	-	-	-	-	-	-	-	*	*
**2**	*	-	-	-	-	-	-	-	-	-	-	-	-	-	-	33.62 ±32.13	*	*
**2.5**	*	-	-	-	-	-	-	-	-	-	-	-	-	-	2.32 ±1.37	17.30 ±18.68	*	*
**3**	*	-	-	-	-	1.29 ±0.28	1.16 ±0.09	1.77 ±0.51	1.20 ±0.05	1.08 ±0.08	1.36 ±0.17	2.03 ±0.33	4.11 ±1.09	1.94 ±0.37	1.50 ±0.07	2.34 ±0.27	*	*
**3.5**	*	1.88 ±0.25	1.42 ±0.27	1.15 ±0.05	1.73 ±0.26	1.20 ±0.04	1.08 ±0.05	1.43 ±0.08	1.14 ±0.04	1.06 ±0.06	1.28 ±0.05	1.72 ±0.40	3.64 ±1.43	1.75 ±0.11	1.43 ±0.06	1.68 ±0.47	*	*
**4**	*	1.76 ±0.70	1.50 ±0.12	1.10 ±0.03	1.52 ±0.49	1.16 ±0.08	1.09 ±0.08	1.39 ±0.18	1.14 ±0.05	1.06 ±0.17	1.17 ±0.04	1.75 ±0.83	4.09 ±3.14	1.40 ±0.33	2.33 ±1.43	*	*	*

Addition computational data

Electronic energy. number of imaginary frequencies. and optimized xyz coordinates for the optimized structure of neutral form of Y**L**$_3$ at the PBE1PBE-D3/def2-TZVP level of theory:

Energy (a.u.) = -3965.0195029

Number of imaginary frequencies: 0

Y	-0.11129	0.07430	0.51819
O	-1.19070	0.78584	-1.30369
O	1.04983	1.98071	0.15243
O	-1.64457	1.25836	1.72372
O	-1.87682	-1.49876	0.60132
O	0.67922	-1.58546	-0.74067
O	1.80713	-0.30047	1.68961
P	3.25367	-0.04644	1.32149
P	1.54319	-2.80703	-0.89971
P	-1.73419	-3.01964	0.44892
P	-3.11679	1.22121	2.00923
P	-1.71071	2.02678	-1.99421
P	2.03733	2.64581	-0.77062
O	2.74885	1.60324	-1.70790
O	1.40526	3.71149	-1.69198
H	0.48113	3.39750	-2.10424
O	3.59338	-0.27577	-0.14366
H	3.10326	0.82512	-1.17393
O	4.16113	-0.99490	2.23660
H	3.72851	-1.83431	2.43481
O	1.67051	-3.61261	0.43044
O	2.98868	-2.46093	-1.36680
H	3.31044	-1.61373	-0.90272
O	-0.66407	-3.62885	1.30970
H	0.73910	-3.65954	0.87882
O	-3.15664	-3.66713	0.79774
H	-3.34648	-3.67949	1.74378
O	-3.72560	-0.18127	1.71603
H	-3.05594	-0.79270	1.24820
O	-3.47006	1.59693	3.50009
H	-3.07166	1.03103	4.17344
O	-0.71984	2.93552	-2.66483
O	-2.72231	1.55359	-3.14173
H	-3.15536	0.72172	-2.91814
C	-2.67509	2.99767	-0.80588
N	-3.85374	2.29895	-0.31682
H	-1.98170	3.19901	0.01495
H	-2.92557	3.96921	-1.25435
C	-4.06424	2.45167	1.10710
H	-5.11095	2.25035	1.34879
H	-3.83168	3.45476	1.49363
C	-5.04243	2.63502	-1.07955
H	-4.85184	2.47955	-2.14080
H	-5.34634	3.68373	-0.93437

H	-5.86745	1.98666	-0.78177
C	3.31212	3.45109	0.23680
N	4.27119	2.57048	0.84872
H	3.84309	4.16971	-0.39208
H	2.77615	4.02419	1.00272
C	3.83769	1.62628	1.83811
C	5.46640	2.25693	0.10499
H	5.86808	3.17427	-0.33142
H	5.32878	1.52419	-0.69907
H	6.21480	1.85739	0.79439
H	3.02559	2.05968	2.42790
H	4.66590	1.43177	2.52864
C	0.74230	-3.84402	-2.14586
N	-0.53198	-4.37856	-1.72924
H	1.40193	-4.66941	-2.42030
H	0.62187	-3.21166	-3.03362
C	-1.58143	-3.47549	-1.33549
H	-1.48705	-2.54055	-1.88981
H	-2.54972	-3.91554	-1.59962
C	-0.54035	-5.69218	-1.13186
H	-0.18734	-5.72306	-0.09371
H	0.08066	-6.36369	-1.72834
H	-1.56293	-6.07859	-1.14827

Electronic energy. number of imaginary frequencies. and optimized xyz coordinates for the optimized structure of zwitterionic form of Y**L**$_3$ at the PBE1PBE-D3/def2-TZVP level of theory:

Energy (a.u.) = -3965.0483065

Number of imaginary frequencies: 0

Y	0.11727	0.34189	0.39548
O	0.00239	-1.27531	1.99852
O	2.24451	0.81726	1.25177
O	-0.76875	-1.19530	-0.96204
O	-2.18604	0.30270	0.79818
O	-0.21632	2.53082	1.41985
O	1.61401	-0.17414	-1.27569
P	2.21103	-1.49386	-1.70196
P	-0.10346	3.26270	0.10450
P	-3.49011	0.95241	0.35513
P	-1.71097	-2.05189	-1.76183
P	-0.12372	-2.77733	1.99472
P	3.42584	1.67344	0.88247
O	3.23980	3.14200	0.68489
O	4.58576	1.40900	1.95380
H	4.28649	0.86067	2.68836
O	1.61865	-2.19134	-2.88382
O	2.43218	-2.45011	-0.45981
H	1.65387	-2.84488	0.05319
O	-0.08776	2.22926	-1.01265
O	1.07021	4.28712	0.05050

H	1.96212	3.85390	0.31529
O	-3.58169	0.88842	-1.22997
H	-3.32313	-0.00219	-1.64333
O	-4.71417	0.51059	1.06195
O	-2.96344	-1.39428	-2.25489
O	-0.92656	-2.77363	-2.91431
H	0.06681	-2.57073	-2.89020
O	0.61940	-3.55098	0.95752
O	0.27424	-3.35465	3.42544
H	0.09554	-2.74168	4.14839
C	-1.90767	-3.18477	1.92119
H	-2.39273	-2.73775	2.79002
H	-2.04997	-4.26510	1.95723
C	-2.14314	-3.34721	-0.55390
H	-2.93370	-4.01910	-0.88129
H	-1.24172	-3.91662	-0.33360
C	-4.06547	-2.70685	0.85064
H	-4.36114	-3.73323	1.05729
H	-4.50393	-2.36346	-0.08374
H	-4.36110	-2.03895	1.65532
C	4.15319	1.05587	-0.67031
H	5.07741	1.58568	-0.89708
H	3.41832	1.26354	-1.44373
C	3.98007	-1.09755	-1.93709
C	5.87126	-0.68715	-0.38158
H	6.13888	-0.15827	0.53042
H	6.46834	-0.33183	-1.21854
H	6.00231	-1.75837	-0.25000
H	4.56415	-2.00768	-2.06322
H	4.15962	-0.43166	-2.77872
C	-1.54200	4.32166	-0.24778
H	-1.29973	4.94825	-1.10628
H	-1.83256	4.95500	0.58866
C	-3.30833	2.76640	0.56583
H	-4.28183	3.22062	0.73696
H	-2.67801	2.93791	1.43668
C	-3.70393	4.15741	-1.42916
H	-4.11088	4.97167	-0.83404
H	-4.48403	3.44672	-1.69173
H	-3.22890	4.54331	-2.32800
H	-2.33593	-1.64918	0.65085
H	-2.30041	2.70142	-1.21996
H	3.88987	-0.84833	0.07388
N	-2.58874	-2.65851	0.70866
N	-2.68474	3.44456	-0.62018
N	4.44180	-0.41634	-0.67692
